# Supplementary material for: Endowing homodimeric carbamoyltransferase GdmN with iterative functions through structural characterization and mechanistic studies
Source: Nat Commun. 2022 Nov 3;13:6617. doi: 10.1038/s41467-022-34387-2 (PMC9633730; doi:10.1038/s41467-022-34387-2)
Supplement: Supplementary file 1 — Supplementary Information [file 41467_2022_34387_MOESM1_ESM.pdf]

# Supplementary Information

## Endowing homodimeric carbamoyltransferase GdmN with iterative functions through structural characterization and mechanistic studies

*Jianhua Wei*[1,2#], *Xuan Zhang*[3#], *Yucong Zhou*[1,2], *Xingnuo Cheng*[1,2], *Zhi Lin*[1,2], *Mancheng Tang*[1,2], *Jianting Zheng*[1,2], *Binju Wang*[3], *Qianjin Kang*[1,2]*, *Linquan Bai*[1,2]*

[1]State Key Laboratory of Microbial Metabolism, Shanghai-Islamabad-Belgrade Joint Innovation Center on Antibacterial Resistances, School of Life Sciences and Biotechnology, Shanghai Jiao Tong University, Shanghai 200240, China.

[2]Joint International Research Laboratory of Metabolic and Developmental Sciences, Shanghai Jiao Tong University, Shanghai 200240, China

[3]State Key Laboratory of Physical Chemistry of Solid Surfaces and Fujian Provincial Key Laboratory of Theoretical and Computational Chemistry, College of Chemistry and Chemical Engineering, Xiamen University, Xiamen 361005, China.

[#]These authors contributed equally: Jianhua Wei, Xuan Zhang.

# Table of Contents

**Supplementary Tables** ........ **1**

Supplementary Table 1 Interactions between chain A and B in the asymmetric unit calculated by PDBePISA. ........ 1

Supplementary Table 2 Data collection and refinement statistics........ 2

Supplementary Table 3 X-ray crystallographic data and structure refinement for 4. ........ 4

Supplementary Table 4 Strains and plasmids used in this work. ........ 5

Supplementary Table 5 PCR primers used in this study. ........ 6

**Supplementary Figures** ........ **8**

Supplementary Figure 1 Various secondary metabolites catalyzed by CTases. ........ 9

Supplementary Figure 2 Schemes for carbamoylation catalyzed by different classes of CTases. ........ 10

Supplementary Figure 3 Phylogenetic analysis and aggregation states of CTases. ........ 11

Supplementary Figure 4 Sequence alignment of different CTases. ........ 12

Supplementary Figure 5 The post-polyketide synthase modification steps of ansamitocin.... 14

Supplementary Figure 6 SDS-PAGE analysis of GdmN and a mutated GdmN with M1-L575 deleted. ........ 15

Supplementary Figure 7 Size-exclusion chromatography and catalytic activities of wild-type GdmN and its mutants........ 16

Supplementary Figure 8 Crystal structures of GdmN. ........ 18

Supplementary Figure 9 Hydrogen-bonding interactions between two chains in the asymmetric unit. ........ 19

Supplementary Figure 10 2*Fo-Fc* map for iron ion and its coordinating residues contoured at 1.0σ. ........ 20

Supplementary Figure 11 Substrate binding pocket of GdmN. ........ 21

Supplementary Figure 12 Clip view of catalytic chamber of GdmN formed by homodimer architecture. ........ 22

Supplementary Figure 13 Superimposition of GdmN structure onto TobZ structure. ........ 23

Supplementary Figure 14 Electrostatic surface potential and interactions with ligands of GdmN and TobZ. ........ 24

Supplementary Figure 15 *Fo-Fc* omit maps for different ligands. ........ 26

Supplementary Figure 16 2*Fo-Fc* maps for different ligands contoured at 1.0 σ. ........ 28

Supplementary Figure 17 Analysis of the YrdC-like domain of GdmN with that of TobZ.... 29

Supplementary Figure 18 *Fo-Fc* omit maps for different ligands. ........ 32

Supplementary Figure 19 2*Fo-Fc* maps for different ligands contoured at 1.0 σ. ........ 34

Supplementary Figure 20 Interaction of ligands with surrounding residues in GdmN complexes structures. ........ 35

Supplementary Figure 21 Crystallographic snapshots during carbamoylation process. ........ 37

Supplementary Figure 22 The QM calculation results for the conversion from 2 to 3. ........ 38

Supplementary Figure 23 B3LYP/6-31G(d)-scanned energy profile (in kcal/mol) for the approach of the amino N atom to the C9 atom of the carbonyl group. ....40
Supplementary Figure 24 Superimposition of the Y82F structure (yellow) onto the GdmN/AMP/ 1 structure (grey) with an r.m.s.d. value of 0.38 Å. ....41
Supplementary Figure 25 The QM/MM calculated mechanisms for the enzymatic reactions catalyzed by GdmN. ....42
Supplementary Figure 26 The overall structure model of Asc21b constructed by SWISS-MODEL server. ....44
Supplementary Figure 27 Proposed residues involved in substrate orientation. ....45
Supplementary Figure 28 The LC-MS analysis of GdmN mutants towards 1 and 3. ....46
Supplementary Figure 29 $^1$H NMR spectrum (a) and $^{13}$C NMR spectrum (b) of 4 in $CDCl_3$. 47
Supplementary Figure 30 HR-ESI-MS analysis of 4. ....48
Supplementary Figure 31 Superimposition of the GdmN V24Y/G157A/R158A/G188R mutant complexed with carbamoyl-AMP (orange) onto the GdmN/carbamoyl-AMP/1 complex (cyan). ....49
Supplementary Figure 32 Structural analysis of the GdmN V24Y/G157A/R158A/G188R mutant and GdmN. ....50
Supplementary Figure 33 Electrostatic surface potential of GdmN and the GdmN V24Y/G157A/R158A/G188R mutant. ....51
Supplementary Figure 34 *Fo-Fc* omit maps (a) and 2*Fo-Fc* maps (b) of carbamoyl-AMP in the Kae1-like domain of the GdmN V24Y/G157A/R158A/G188R mutant in complex with carbamoyl-AMP (PDB: 7VZQ). ....52
Supplementary Figure 35 Binding position of carbamoyl-AMP in the GdmN V24Y/G157A/R158A/G188R mutant complexed with carbamoyl-AMP and the GdmN/carbamoyl-AMP/1 complex. ....53
Supplementary Figure 36 Computational docking of 3 into the GdmN V24Y/G157A/R158A/G188R mutant structure. ....55
Supplementary Figure 37 The MD simulations of the wild-type GdmN with 3. ....56
Supplementary Figure 38 The MD simulations of the tetra-mutant GdmN with 3. ....56
Supplementary Figure 39 Monomer models, structural superposition, and sequence alignment for substrate binding of Asm21, Asc21b, NovN, and Orf7*. ....57
Supplementary Figure 40 Schematic construction and PCR validation of WJH10. ....59
Supplementary Figure 41 Schematic construction and PCR validation of WJH13. ....60
Supplementary Figure 42 Schematic construction and PCR validation of WJH11. ....61
Supplementary Figure 43 Schematic construction and PCR validation of WJH12. ....62
**Supplementary References ....63**

# Supplementary Tables

## Supplementary Table 1 Interactions between chain A and B in the asymmetric unit calculated by PDBePISA.

| Chain A | Chain B | distance (Å) |
|---|---|---|
| Tyr96 | Arg260 | 2.81 |
| Tyr99 | Arg260 | 3.04 |
| Pro100 | Lys261 | 3.00 |
| Ala102 | Arg260 | 2.72 |
| Arg158 | Ile241 | 2.87 |
| Ile241 | Arg158 | 2.72 |
| Arg260 | Tyr96 | 2.58 |
| Arg260 | Ala102 | 3.05 |
| Arg260 | Ala102 | 3.05 |
| Lys261 | Pro100 | 2.97 |
| Phe582 | Ala598 | 2.78 |
| Thr586 | Ala594 | 3.77 |
| Leu588 | Arg592 | 2.83 |
| Leu588 | Arg592 | 2.99 |
| Glu590 | Glu590 | 2.75 |
| Glu590 | Tyr624 | 3.84 |
| Glu590 | Glu590 | 2.80 |
| Glu590 | Tyr624 | 2.50 |
| Arg592 | Leu588 | 2.73 |
| Arg592 | Gly628 | 3.68 |
| Arg592 | Leu588 | 2.85 |
| Ala594 | Thr586 | 3.51 |
| Ala598 | Phe582 | 3.03 |
| Ala600 | Val630 | 2.83 |
| Tyr624 | Glu590 | 3.75 |
| Tyr624 | Glu590 | 2.63 |
| Gly628 | Arg592 | 3.35 |
| Val630 | Ala600 | 2.99 |

**Supplementary Table 2 Data collection and refinement statistics.**

Values in parentheses are for the highest resolution shell.

| dataset | GdmN | The structure of GdmN with ATP | The structure of GdmN with carbamoyl-AMP | GdmN with AMP and **1** | GdmN with carbamoyl-AMP and **1** |
|---|---|---|---|---|---|
| **Data collection** | | | | | |
| Space group | $P3_121$ | $P3_121$ | $P3_121$ | $P3_121$ | $P3_121$ |
| Cell dimensions | | | | | |
| *a, b, c* (Å) | 110.955, 110.955, 231.544 | 111.717, 111.717, 230.717 | 110.531, 110.531, 231.460 | 111.483, 111.483, 231.355 | 111.394, 111.394, 230.892 |
| *α, β, γ* (°) | 90, 90, 120 | 90, 90, 120 | 90, 90, 120 | 90, 90, 120 | 90, 90, 120 |
| Resolution (Å) | 40.00 - 2.25 (2.33 - 2.25) | 50.00 - 2.00 (2.03 - 2.00) | 50.00 - 1.98 (2.01 - 1.98) | 50.00 - 2.80 (2.85 - 2.80) | 50.00 -2.10 (2.14 - 2.10) |
| $R_{merge}$ | 0.072 (0.485) | 0.104 (1.025) | 0.087 (1.046) | 0.117 (0.625) | 0.107 (0.798) |
| $R_{pim}$ | 0.024 (0.163) | 0.024 (0.244) | 0.029 (0.353) | 0.030 (0.165) | 0.024 (0.179) |
| $I / \sigma I$ | 32.533 (5.000) | 36.368 (4.214) | 27.737 (2.429) | 27.000 (5.941) | 34.636 (5.444) |
| Completeness (%) | 100.0 (100.0) | 100.0 (100.0) | 100.0 (100.0) | 100.0 (100.0) | 100.0 (100.0) |
| Redundancy | 9.4 (9.5) | 18.7 (18.4) | 9.7 (9.7) | 15.8 (15.1) | 19.9 (20.7) |
| $CC_{1/2}$ | 0.998 (0.961) | 0.998 (0.932) | 0.998 (0.858) | 0.995 (0.966) | 0.999 (0.956) |
| **Refinement** | | | | | |
| Resolution (Å) | 36.970 - 2.251 (2.332 - 2.251) | 25.635 - 2.001 (2.072 - 2.001) | 36.881 - 1.982 (2.053 - 1.982) | 31.875 - 2.806 (2.906 - 2.806) | 28.617 - 2.101 (2.176 - 2.101) |
| No. reflections | 78811 (7705) | 112180 (10535) | 111707 (9014) | 41478 (4068) | 97138 (9510) |
| $R_{work}$ / $R_{free}$ | 0.1761/0.2089 (0.2051/0.2477) | 0.1822/0.2115 (0.2262/0.2694) | 0.1772/0.2071 (0.2356/0.2843) | 0.1707/0.2202 (0.2481/0.3562) | 0.1833/0.2118 (0.2121/0.2546) |
| No. atoms | | | | | |
| Protein | 10419 | 10446 | 10460 | 10438 | 10408 |
| Ligand | 121 | 195 | 166 | 196 | 212 |
| Water | 289 | 445 | 663 | / | 355 |
| *B*-factors (Å$^2$) | | | | | |
| Protein | 32.04 | 26.95 | 28.05 | 37.25 | 26.38 |
| Ligand | 46.27 | 37.47 | 36.61 | 46.56 | 31.53 |
| Water | 30.36 | 27.53 | 32.35 | / | 25.67 |
| **R.m.s. deviations** | | | | | |
| bond lengths (Å) | 0.008 | 0.007 | 0.008 | 0.009 | 0.007 |
| bond angles (˚) | 0.89 | 0.87 | 0.95 | 1.06 | 0.86 |
| Ramachandran outliers (%) | 0.00 | 0.00 | 0.07 | 0.07 | 0.00 |
| Ramachandran favored (%) | 97.69 | 97.70 | 97.48 | 96.14 | 97.33 |
| **PDB code** | **7VYO** | **7VX0** | **7VYJ** | **7VZY** | **7VZN** |

| dataset | GdmN with the natural tetrahedral intermediate, carbamoyl-AMP, and **1** | GdmN with the natural tetrahedral intermediate, AMP, and **2** | GdmN Y82F mutant | GdmN V24Y/G157A/R158A/G188R mutant with carbamoyl-AMP |
|---|---|---|---|---|
| **Data collection** | | | | |
| Space group | $P3_121$ | $P3_121$ | $P3_121$ | $P3_121$ |
| Cell dimensions | | | | |
| *a, b, c* (Å) | 111.860, 111.860, 231.272 | 111.029, 111.029, 231.276 | 111.692, 111.692, 231.408 | 111.092, 111.092, 231.802 |
| *α, β, γ* (°) | 90, 90, 120 | 90, 90, 120 | 90, 90, 120 | 90, 90, 120 |
| Resolution (Å) | 50.00 - 2.85 (2.90 - 2.85) | 50.00 - 2.88 (2.93 - 2.88) | 50.00 - 2.30 (2.34 - 2.30) | 50.00 - 2.10 (2.14 - 2.10) |
| $R_{merge}$ | 0.118 (0.500) | 0.129 (0.548) | 0.105 (0.388) | 0.150 (1.064) |
| $R_{pim}$ | 0.031 (0.131) | 0.043 (0.180) | 0.025 (0.094) | 0.035 (0.257) |
| $I / \sigma I$ | 27.600 (9.333) | 21.167 (5.333) | 29.188 (7.182) | 18.875(2.200) |
| Completeness (%) | 100.0 (100.0) | 100.0 (100.0) | 100.0 (100.0) | 100.0 (100.0) |
| Redundancy | 15.2 (15.2) | 9.8 (10.1) | 17.5 (16.4) | 19.8 (17.8) |
| $CC_{1/2}$ | 0.996 (0.982) | 0.989 (0.973) | 0.997 (0.983) | 0.989 (0.910) |
| **Refinement** | | | | |
| Resolution (Å) | 25.697 - 2.853 (2.955 - 2.853) | 27.757 - 2.881 (2.984 - 2.881) | 35.825 - 2.300 (2.382 - 2.300) | 44.429 - 2.104 (2.179 - 2.104) |
| No. reflections | 39635 (3853) | 38031 (3737) | 74834 (7286) | 96338 (9254) |
| $R_{work}$ / $R_{free}$ | 0.1753/0.2252 (0.2164/0.2879) | 0.1660/0.2156 (0.1924/0.2840) | 0.1824/0.2268 (0.2197/0.3013) | 0.1964-0.2332 (0.2687-0.3493) |
| No. atoms | | | | |
| Protein | 10407 | 10338 | 10400 | 10426 |
| Ligand | 209 | 210 | 51 | 127 |
| Water | 16 | 70 | 292 | 261 |
| *B*-factors ($Å^2$) | | | | |
| Protein | 37.87 | 43.70 | 30.05 | 40.15 |
| Ligand | 46.26 | 48.91 | 42.29 | 45.69 |
| Water | 31.74 | 36.48 | 27.64 | 36.39 |
| **R.m.s. deviations** | | | | |
| bond lengths (Å) | 0.010 | 0.009 | 0.008 | 0.007 |
| bond angles (°) | 1.01 | 1.03 | 0.89 | 0.90 |
| Ramachandran outliers (%) | 0.00 | 0.30 | 0.00 | 0.00 |
| Ramachandran favored (%) | 96.36 | 95.16 | 97.55 | 96.89 |
| **PDB code** | **7VZZ** | **7VYP** | **7VZU** | **7VZQ** |

**Supplementary Table 3 X-ray crystallographic data and structure refinement for 4.**

Crystallographic data for the structure of **4** have been deposited to the Cambridge Crystallographic Data Center with accession number CCDC 2122658. There are two B-level alerts during checkcif procedure, which didn't influence X-ray crystallographic analysis of **4**.

| | |
|---|---|
| Identification code | t_a |
| Empirical formula | C28 H40 Cl N3 O10 |
| Chemical formula moiety | C28 H36 Cl N3 O8, 2 (H2 O) |
| Formula weight | 614.08 |
| Temperature | 173(2) K |
| Wavelength | 1.54178 Å |
| Crystal system | Orthorhombic |
| Space group | P2(1)2(1)2(1) |
| Unit cell dimension | a = 10.1130(2) Å α = 90°. |
| | b = 11.5120(2) Å β = 90°. |
| | c = 26.3931(5) Å γ = 90°. |
| Volume | 3072.71(10) $Å^3$ |
| Z | 4 |
| Density (calculated) | 1.327 $Mg/m^3$ |
| Absorption coefficient | 1.607 $mm^{-1}$ |
| F(000) | 1304 |
| Crystal size | 0.160 x 0.150 x 0.140 mm |
| Theta range for data collection | 3.349 to 68.258°. |
| Limiting indices | -12<=h<=12, -13<=k<=13, -31<=l<=31 |
| Reflections collected / unique | 25258 / 5626 [R(int) = 0.0438] |
| Completeness to theta = 67.679 | 100.0 % |
| Absorption correction | Semi-empirical from equivalents |
| Max. and min. transmission | 0.7531 and 0.6319 |
| Refinement method | Full-matrix least-squares on $F^2$ |
| Data / restraints / parameters | 5626 / 2 / 410 |
| Goodness-of-fit on $F^2$ | 1.059 |
| Final R indices [I>2sigma(I)] | R1 = 0.0292, wR2 = 0.0771 |
| R indices (all data) | R1 = 0.0327, wR2 = 0.0793 |
| Absolute structure parameter | 0.007(7) |
| Extinction coefficient | n/a |
| Largest diff. peak and hole | 0.229 and -0.203 $e.A^{-3}$ |

**Supplementary Table 4 Strains and plasmids used in this work.**

| Strains and plasmids | Features | Source |
|---|---|---|
| ***Streptomyces hygroscopicus* XM201** | Wild-type strain for geldanamycin | [1] |
| ***Actinosynnema pretiosum* subsp. *pretiosum* ATCC 31280** | Wild-type strain for ansamitocin P-3 | ATCC |
| WJH10 | ATCC 31280*Δansa23* | This work |
| WJH11 | WJH10::pLQ1053 | This work |
| WJH12 | WJH10::pLQ1054 | This work |
| WJH13 | ATCC 31280*Δansa21* | This work |
| ***Escherichia coli*** | | |
| DH10B | $F^-$ *mcrA* Δ(mrr-*hsd*RMS-*mcr*BC) φ80*lacZ*ΔM15 Δ*lac*X74 *rec*A1 *end*A1 *ara*D139 Δ (*ara*, *leu*)7997 *gal*E15 *gal*K λ - *rsp*L *nup*G | Invitrogen |
| ET12567 (pUZ8002) | $F^-$ dam-13::Tn9 dcm-6 *hsd*M *hsd*R zjj-202::Tn10 *rec*F143 *gal*K2 *gal*T22 *ara*-14 *lac*Y1 xyl-5 *leu*B6 thi-1 *ton*A31 *rps*L136 hisG4 tsx-78 mtl-1 *gln*V44 $Cml^R$ $Kan^R$ | [2] |
| BL21(DE3)pLysS | $F^-$ *ompT* *hsdS*$_B$($r_{B-}$ $m_{B-}$) gal dcm(DE3) pLysS (CamR) | Sangon Biotech |
| **Plasmids** | | |
| pBluescript SK(+) | *ori* (f1), *ori* (pUC18), *bla*, *lacZ* | [3] |
| pJTU1278 | *rep-pIJ101, tsr, oriT* | [4] |
| pLQ1051 | Construction for *ansa23* inactivation | This work |
| pLQ1052 | Construction for *ansa21* inactivation | This work |
| pSET152 | *ΦC31 int, aac(3)IV lacZα* $oriT_{RK2}$ | [2] |
| pLQ646 | pSET152::*kasO*p* | [5] |
| pLQ1053 | pLQ646::*gdmN* | This work |
| pLQ1054 | pLQ646::*gdmNm (*truncated *gdmN*, M1-L575) | This work |
| pET28a | *kan*, $P_{T7}$, $His_6$-tag | Novagen |
| pLQ1055 | pET28a with *gdmN* | This work |
| pLQ1056 | pET28a with *gdmNm* | This work |

**Supplementary Table 5 PCR primers used in this study.**

| Primers | SEQUENCE (from 5′ to 3′) | |
|---|---|---|
| *ansa23*-deletion-L-FP | AAAGGATCCACAGGTCAGATCCAGAGCAGG | *Bam*HI |
| *ansa23*-deletion-L-RP | AAAGAATTCACGAGGTCTTCCTGGACTACG | *Eco*RI |
| *ansa23*-deletion-R-FP | AAAGAATTCTGGTCCTCGGGGAAGTAGAACG | *Eco*RI |
| *ansa23*-deletion-R-RP | AAAAAGCTTAGGTGGTGTTCTACCTGCTGCTGC | *Hind*III |
| *ansa21*-deletion-L-FP | AAAGGATCCAGCACAGCGACTCGAACACC | *Bam*HI |
| *ansa21*-deletion-L-RP | AAAGAATTCCTACGCGACGTACCTGCTGCACTGG | *Eco*RI |
| *ansa21*-deletion-R-FP | AAAGAATTCAGCACGAAGAACAACGACAACG | *Eco*RI |
| *ansa21*-deletion-R-RP | AAAAAGCTTTCAGGGTGTGCGAGGAGACCAGG | *Hind*III |
| *ansa23*-deletion-verify-FP | TGCTGCACTGCGCCACACCCACG | |
| *ansa23*-deletion-verify-RP | TTCGTGGGGGTGTCAATCGCTTGTGG | |
| *ansa21*-deletion-verify-FP | TCACCGACAACGACCGCTACCTGG | |
| *ansa21*-deletion-verify-RP | ACGTCGCCTACCTGACCATCC | |
| *apr*-verify-FP | AATACGAATGGCGAAAAGC | |
| *apr*-verify-RP | ATCGCATTCTTCGCATCC | |
| *gdmN*-FP | AAACATATGCTTGTGCTCGGGCTCAAC | *Nde*I |
| *gdmN*-RP | AAAGAATTCTCAGTGCCCCCGCGTACCGTC | *Eco*RI |
| GdmNm (mutant of C-terminal deletion)-FP | AAACATATGCTTGTGCTCGGGCTCAA) | *Nde*I |
| GdmNm (mutant of C-terminal deletion)-RP | AAAGAATTCTCACAGGTCGGGCGAGGCT | *Eco*RI |
| mutant of deletion of 586-597-FP | CGCCCGGTGGCCTCGGCGGGAGCCAAGACC | |
| mutant of deletion of 586-597-RP | CGCCGAGGCCACCGGGCGGAACCGCGGCAC | |
| mutant of deletion of 586-609-FP | CGCCCGGTGGACTACGACGGCGGCCCGTCC | |
| mutant of deletion of 586-609-RP | GTCGTAGTCCACCGGGCGGAACCGCGGCAC | |
| H87A-FP | GAGAACGCGATCGACACCGTCCTCAACCAC | |
| H87A-RP | GTCGATCGCGTTCTCGGGAAAGTAGTAGCC | |
| H87T-FP | GAGAACACCATCGACACCGTCCTCAACCAC | |
| H87T-RP | GTCGATGGTGTTCTCGGGAAAGTAGTAGCC | |
| R158A-FP | GACGGCGCTGGCGAACTGCACTCCGGCACC | |
| R158A-RP | TTCGCCAGCGCCGTCCAGCACCAGGACCAG | |
| R158M-FP | GACGGCATGGGCGAACTGCACTCCGGCACC | |
| R158M-RP | TTCGCCCATGCCGTCCAGCACCAGGACCAG | |
| R158Q-FP | GACGGCCAGGGCGAACTGCACTCCGGCACC | |
| R158Q-RP | TTCGCCCTGGCCGTCCAGCACCAGGACCAG | |
| M242A-FP | AACATCGCGGTGCCGAACCTGGTCAGCCCG | |
| M242A-RP | CGGCACCGCGATGTTGCCGTGCAGCTCGTA | |
| M242F-FP | AACATCTTCGTGCCGAACCTGGTCAGCCCG | |

| | |
|---|---|
| M242F-RP | CGGCACGAAGATGTTGCCGTGCAGCTCGTA |
| M242-D3-FP | CACGGCAACCCGAACCTGGTCAGCCCGCTG |
| M242-D3-RP | CAGGTTCGGGTTGCCGTGCAGCTCGTACTC |
| M242-D7-FP | GAGCTGCACCTGGTCAGCCCGCTGTTCTAC |
| M242-D7-RP | GCTGACCAGGTGCAGCTCGTACTCGCCGTT |
| H27N-FP | CGAGTCGTTAAAGAACACCTCTCCGAGCTG |
| H27N-RP | TTCTTTAACGACTCGGCGGCTTCCTTGATC |
| Y82F-FP | GGCTACTTCTTTCCCGAGAACCACATCGAC |
| Y82F-RP | GGGAAAGAAGTAGCCCACCGCGTCGACGTC |
| K443A-FP | GCGCTTCGCCACCATCGCGTTGATGCGGGT |
| K443A-RP | ATGGTGGCGAAGCGCGAGGGCTTCCGGCCG |
| M476I-FP | GAAGGAGATGAACTCGTGGTTGCCATCCGC |
| M476I-RP | GAGTTCATCTCCTTCGTGGTGCCGGTGCTG |
| S533A-FP | GTTGAACGCGGTGTTGAGGAGCACGGGGGT |
| S533A-RP | AACACCGCGTTCAACAACAACGCCGAACCC |
| V24Y-FP | GGAGAGTACTTCTTTCATGACTCGGCGGCT |
| V24Y-RP | AAAGAAGTACTCTCCGAGCTGCGGCACCAC |
| G157A/R158A-FP | GTGCTGGACGCGGCGGGCGAACTGCACTCCGGC |
| G157A/R158A-RP | CAGTTCGCCCGCCGCGTCCAGCACCAGGACCAG |
| G188R-FP | CTCGGCCGTCTCTACCTGAACGCCACCTAT |
| G188R-RP | GTAGAGACGGCCGAGCGACTTGGGCACCGG |

FP stands for forward primer, and RP stands for reverse primer.

# Supplementary Figures

a

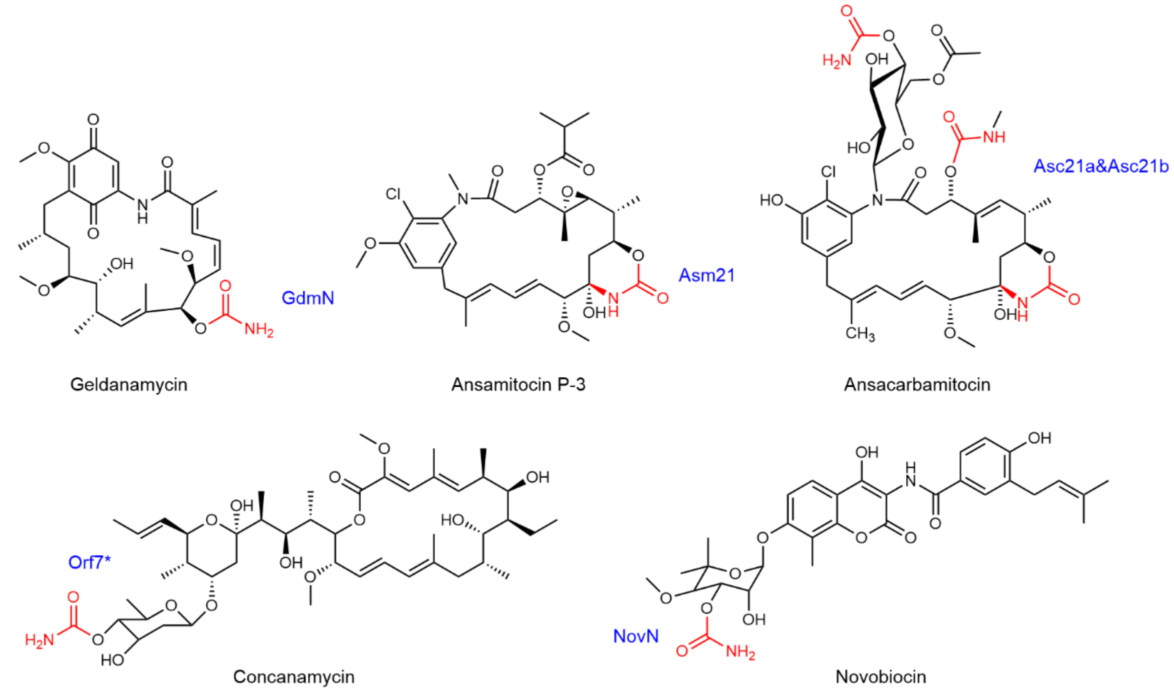


b

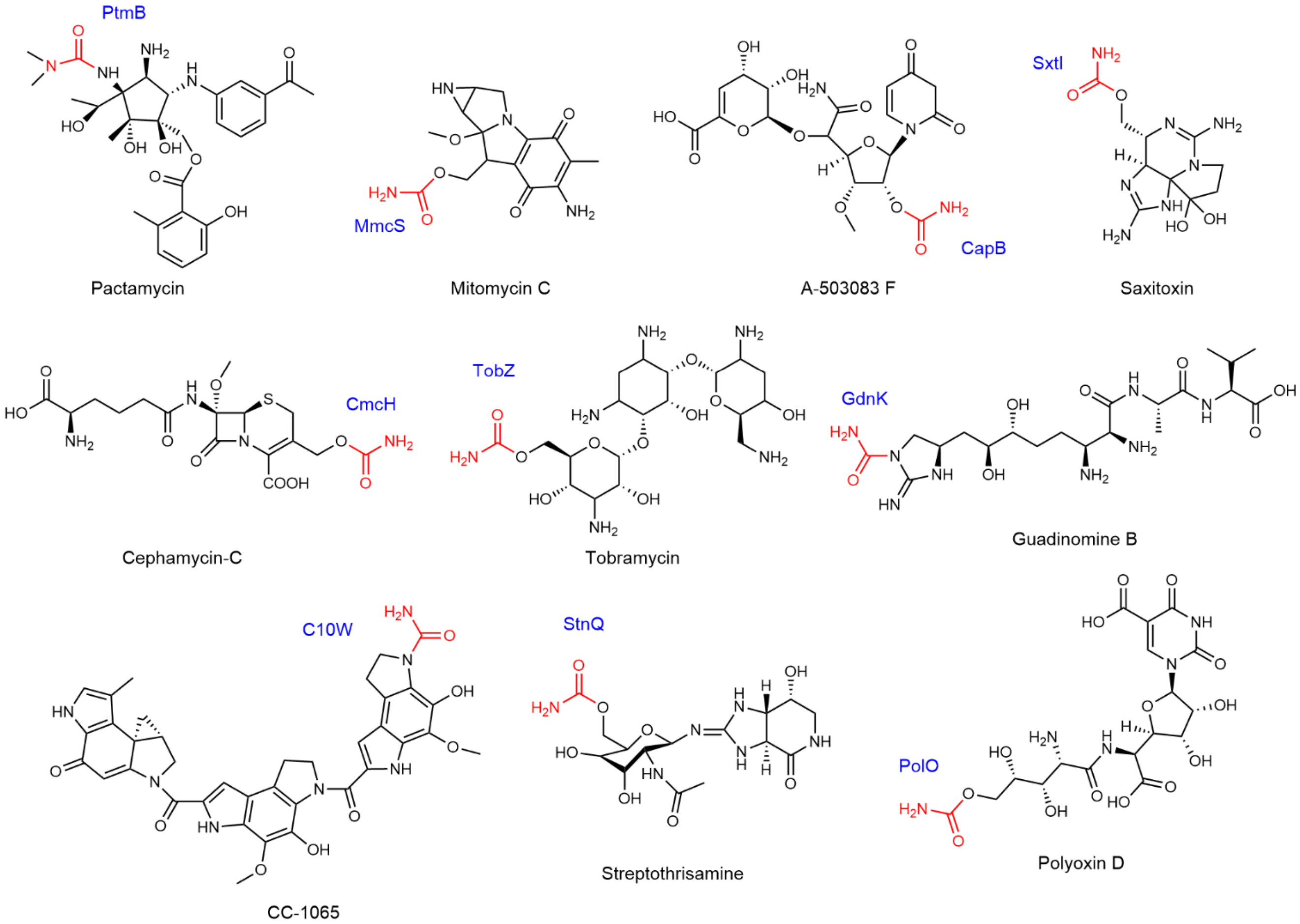

**Supplementary Figure 1 Various secondary metabolites catalyzed by CTases.**

**(a**) Secondary metabolites catalyzed by GdmN-type CTases. **(b)** Secondary metabolites catalyzed by other class III CTases. The names of CTases are in blue and are located next to the carbamoyl group of each molecule.

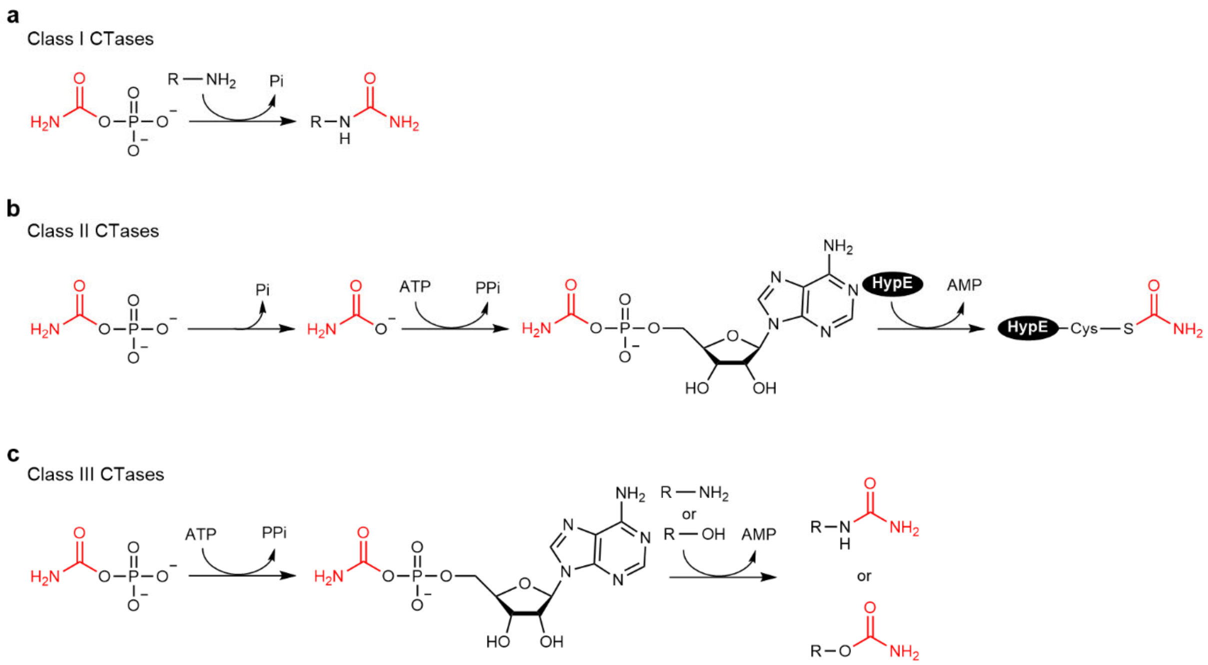


**Supplementary Figure 2 Schemes for carbamoylation catalyzed by different classes of CTases.**

**(a) Class I** CTases, represented by aspartate transcarbamylase (ATCase) and ornithine transcarbamylase (OTCase), which directly transfer carbamoyl group from CP to the recipient substrates. **(b) Class II** CTases, represented by the [NiFe]-hydrogenase maturation factor HypF, which stalls carbamoyl group onto the thiol of C-terminal cysteine of HypE via three half-reactions: HypF first generates carbamate derived from CP, followed by carbamoyladenylate (carbamoyl-AMP) formation in the presence of ATP and $Mg^{2+}$, and then it transfers the carbamoyl group from carbamoyl-AMP to the thiol of the C-terminal cysteine of HypE. **(c) Class III** CTases, which exhibit two half-reactions: enzymes generate carbamoyl-AMP in the presence of CP, ATP and $Mg^{2+}$ and further transfer carbamoyl group to secondary metabolites. According to the reactive groups, carbamoylation catalyzed by class III CTases can be divided into *N*-carbamoylation and *O*-carbamoylation.

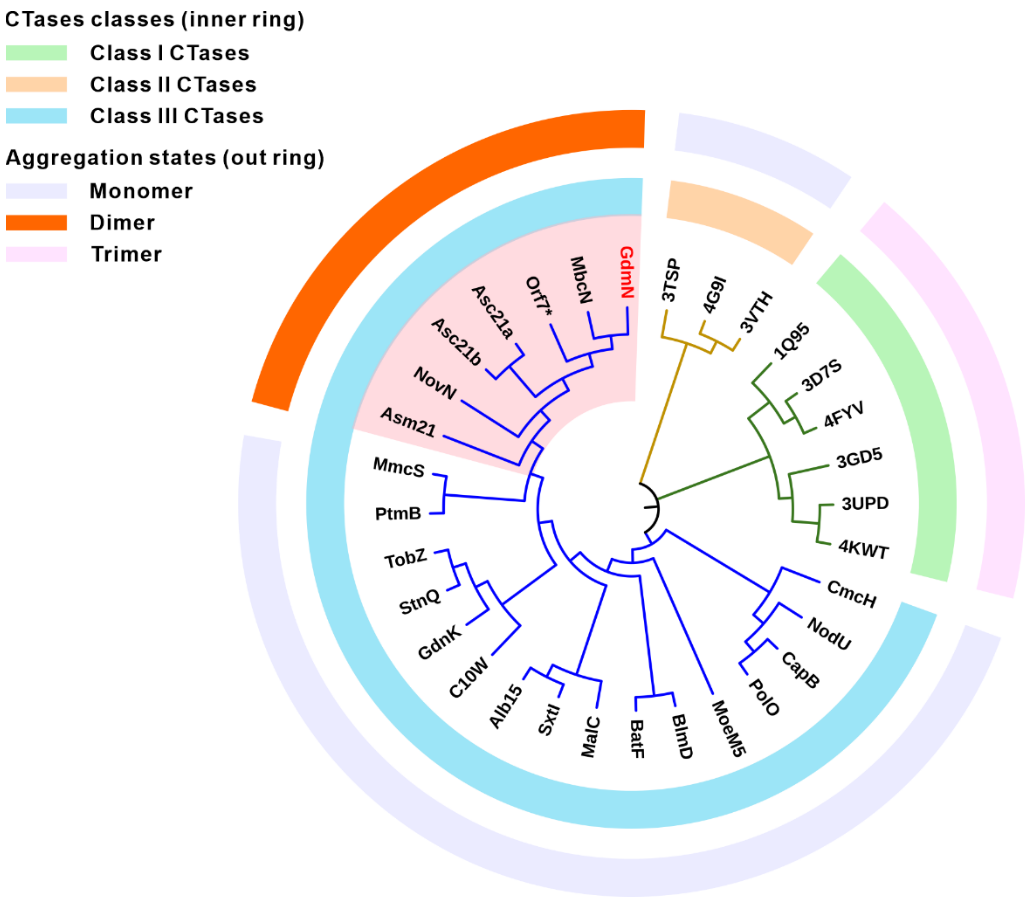


**Supplementary Figure 3 Phylogenetic analysis and aggregation states of CTases.**

Branches are colored based on CTases classes. GdmN-type CTases are indicated in pink, and GdmN is shown in red. Class I CTases contain ATCases from *Escherichia coli* (PDB: 1Q95), ATCases from *Escherichia coli* K-12 (PDB: 3D7S and 4FYV), OTCases from *Gloeobacter violaceus* (PDB: 3GD5), OTCases from *Vibrio vulnificus* (PDB: 3UPD and 4KWT) and so on. Class II CTases contain HypF from *Escherichia coli* (PDB: 3TSP), HypF from *Thermococcus kodakarenesis* (PDB: 4G9I), HypF from *Caldanaerobacter subterraneus* (PDB: 3VTH) and so on.

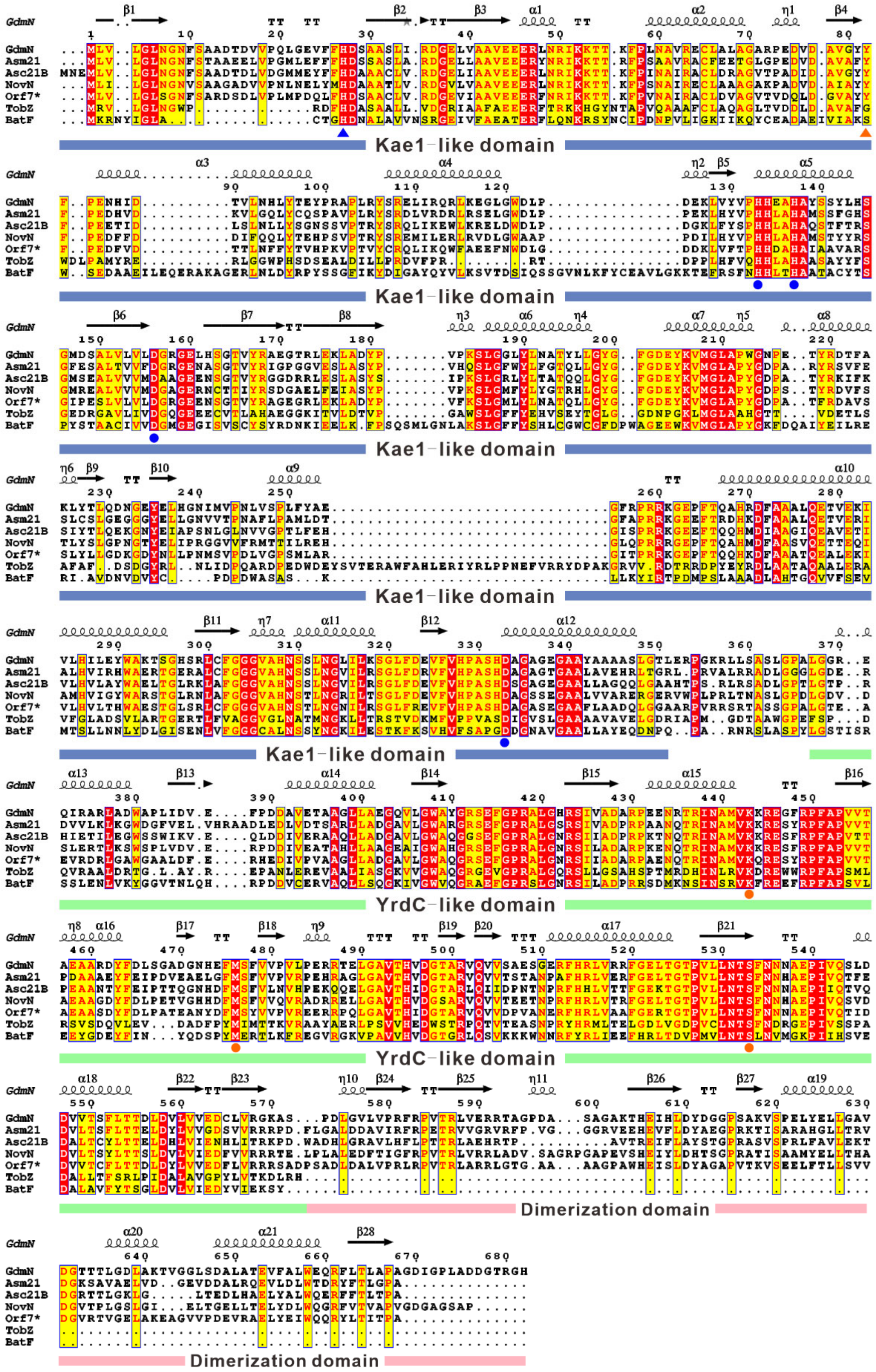


**Supplementary Figure 4 Sequence alignment of different CTases.**

Sequence alignment result of GdmN, Asm21, Asc21b, NovN, Orf7*, TobZ and BatF

revealed that GdmN-type CTases contain an unusual dimerization domain (colored in pink) at the C terminus. The sequence of Kae1-like domain is colored in violet, and the sequence of YrdC-like domain is colored in green. The active site histidine (H27 in GdmN) is marked with a blue triangle. The “keystone” tyrosine, which stabilizes the substrate binding pocket (Y82 in GdmN), is marked with an orange triangle. Highly conserved two histidines and two aspartates responsible for coordinating iron ion (H133, H137, D156, and D333 in GdmN) are marked with blue circles. The residues responsible for generation of carbamoyl-AMP (K443, M476, and S533 in GdmN) are marked with orange circles.

The sequence alignment was performed by T-coffee web-server with default parameters[6]. The result was performed by ESPript 3.0. Residues with strict identity are colored in white against a red background, and those residues with above 70% identity are colored in red within blue frames[7].

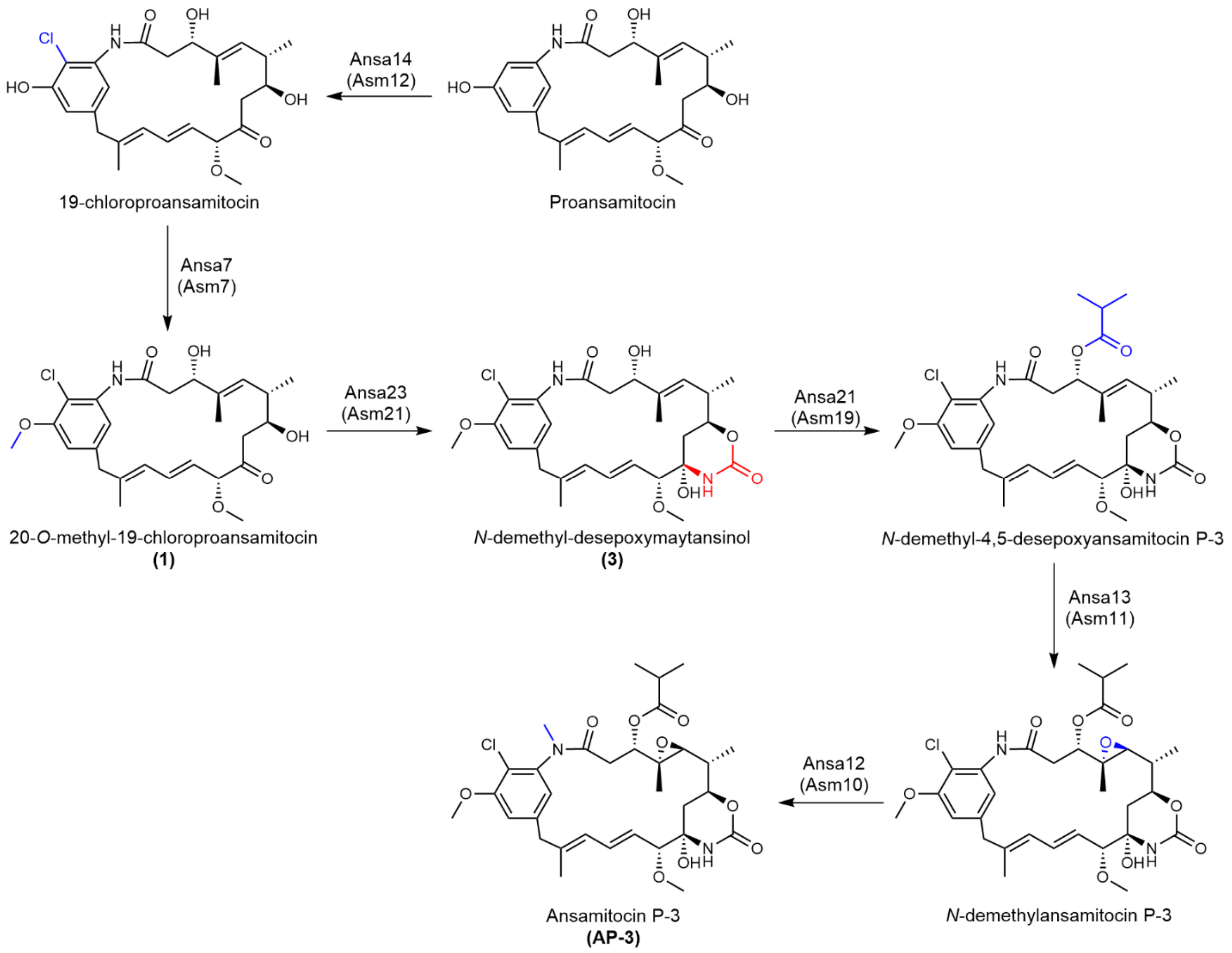


**Supplementary Figure 5 The post-polyketide synthase modification steps of ansamitocin.**

Ansa23, a homologous protein of Asm21, encoded by gene *ansa23* in *A. pretiosum* ATCC 31280 is responsible for the carbamoylation of C-7 hydroxy group of ansamitocin P-3. Compound **1** was accumulated in WJH10 (a mutant strain with gene *ansa23* inactivation). Ansa21, a homologous protein of Asm19, encoded by gene *ansa21* acts as the acyltransferase attaching ester side chain to the C-3 hydroxy group. Compound **3** was accumulated in WJH13 (a mutant strain with gene *ansa21* inactivation).

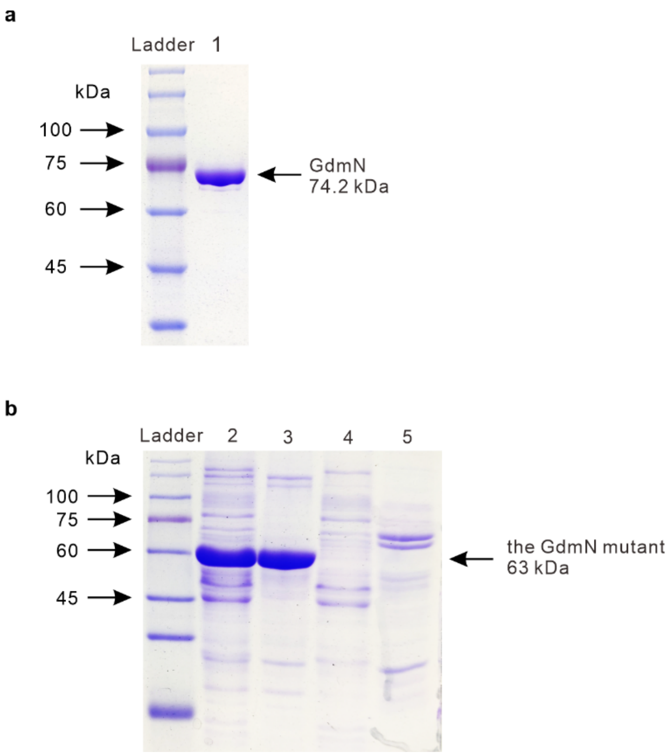


**Supplementary Figure 6 SDS-PAGE analysis of GdmN and a mutated GdmN with M1-L575 deleted.**

**(a)** SDS-PAGE analysis of GdmN. Lane ladder: Protein ladder; Lane 1: Purified GdmN. **(b)** SDS-PAGE analysis of the mutated GdmN with M1-L575 deleted. Lane ladder: Protein ladder; Lane 2: Cell lysate of the mutated GdmN with M1-L575 deleted; Lane 3: Precipitation of the mutated GdmN with M1-L575 deleted; Lane 4: Supernatant of the mutated GdmN with M1-L575 deleted; Lane 5: Elution of the mutated GdmN with M1-L575 deleted. The predicted molecular weight of GdmN is 74.2 kDa, and the predicted molecular weight of the mutated GdmN with M1-L575 deleted is 63 kDa. The experiments were repeated for three times with similar results.

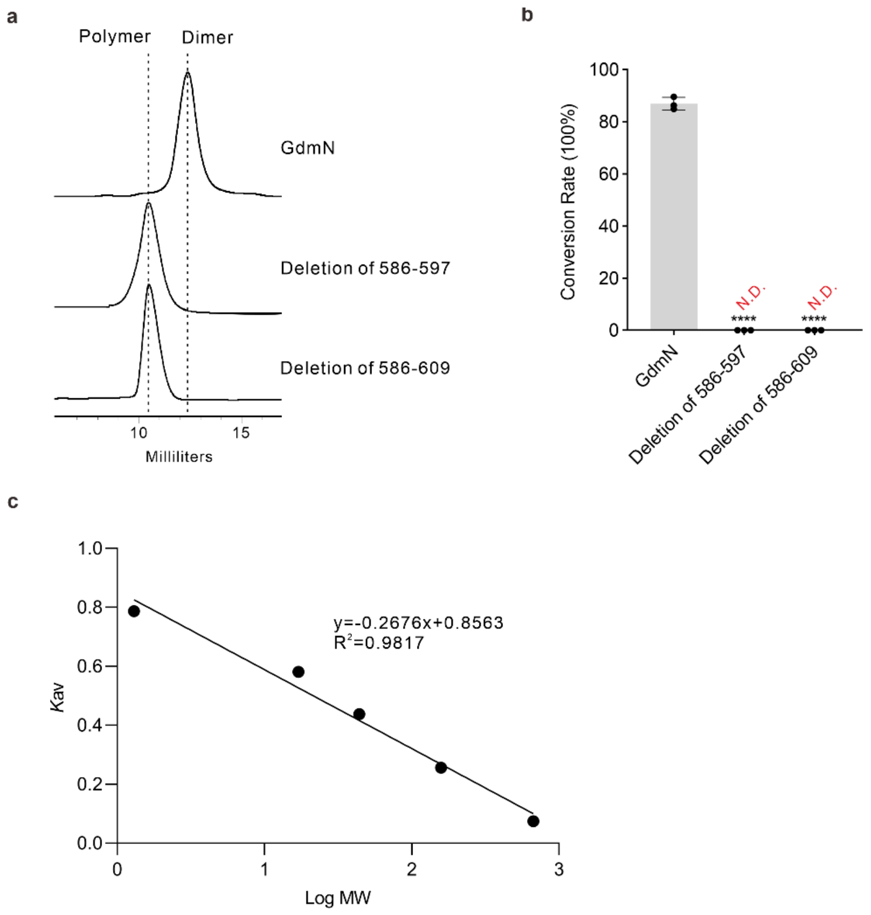


**Supplementary Figure 7 Size-exclusion chromatography and catalytic activities of wild-type GdmN and its mutants.**

**(a)** Size-exclusion chromatography of wild-type GdmN and mutants. GdmN eluted at retention volumes of 12.4 ml, correlating to a molecular weight (MW) of 148.7 kDa. The calculated MW for GdmN is ~74.2 kDa. Thus, GdmN forms a dimer in solution. The mutated GdmN eluted at almost same retention volumes of 10.4 ml, correlating to a MW of 435.9 kDa. The calculated MWs for the mutated GdmN with a deletion of T586-D597 and T586-L609 are 72.9 kDa and 71.7 kDa, respectively. Thus, the mutated GdmN might form polymers with no biological activities in solution. The experiments were repeated for three times with similar results. **(b)** Catalytic activities of wild-type

GdmN and mutants. The activity assays of GdmN and mutants were performed with **1**, CP, ATP, and $Mg^{2+}$ at 30 °C for 12 h. N.D., products not detected. Wild-type GdmN was used as the control. Graphs depict means ± SD (n = 3 independent experiments). Statistical analysis was performed by one-way ANOVA with Dunnett's multiple-comparison test (*****P* < 0.0001). Source data are provided as a Source Data file. **(c)** Calibration curve of size-exclusion chromatography.

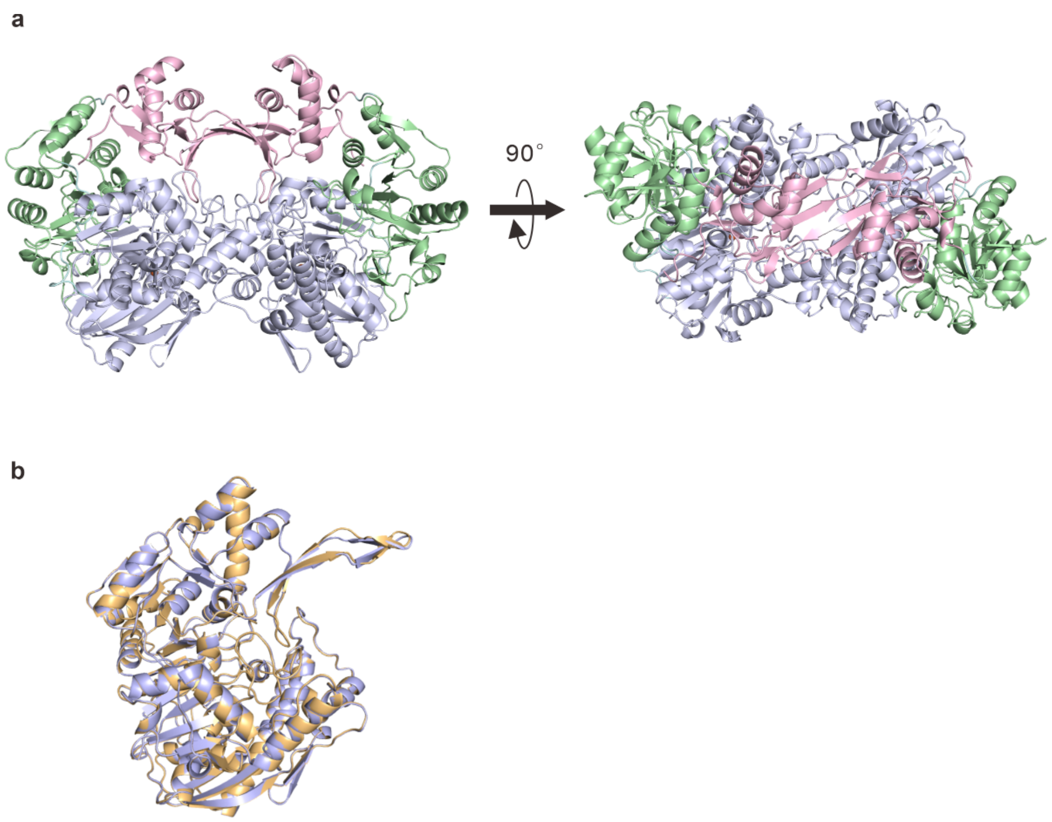


**Supplementary Figure 8 Crystal structures of GdmN.**

**(a)** The overall structure of GdmN. GdmN forms a homodimer architecture in the asymmetric unit. Two bow-shaped monomers complement each other in a face-to-face fashion. Kae1-like domain, YrdC-like domain, and dimerization domain are colored in violet, green, and pink, respectively. Linkers are colored in cyan.

**(b)** The superposition of the 2 subunits of GdmN in the asymmetric unit, with different monomers colored in orange and violet.

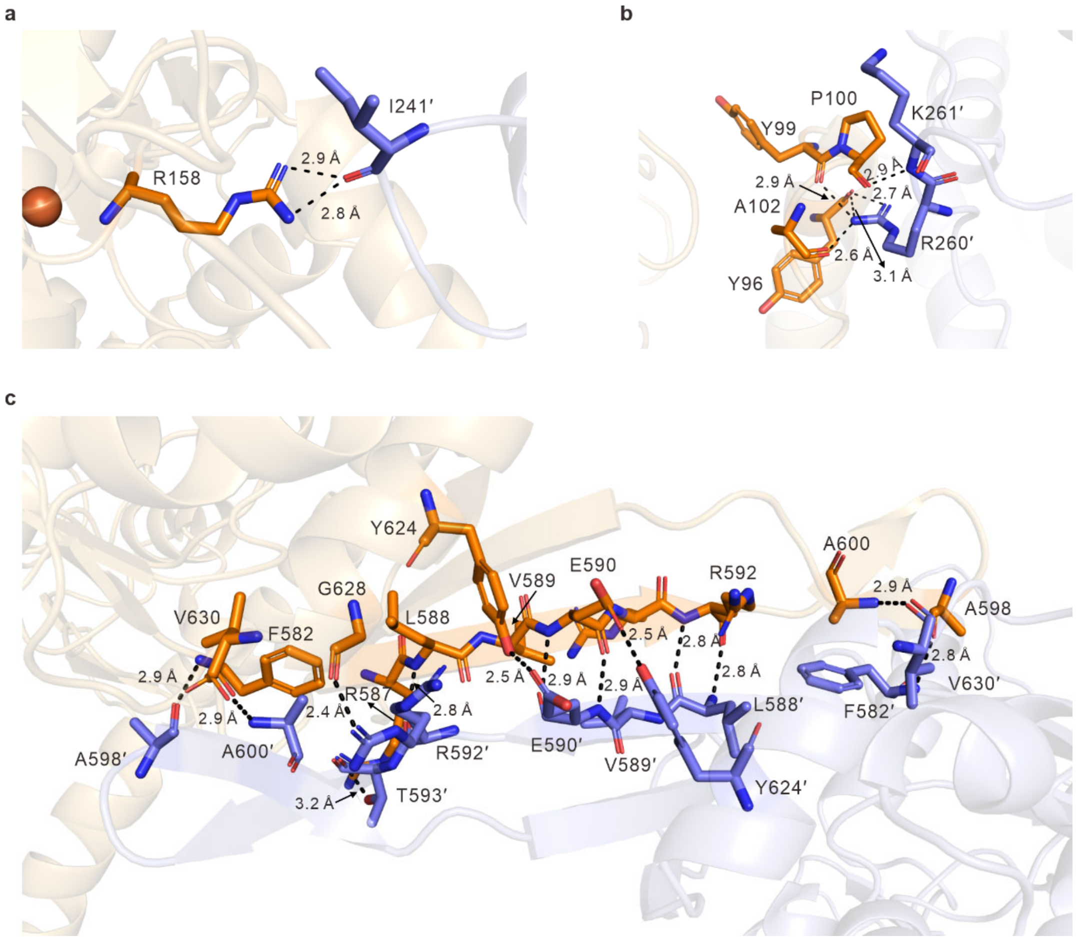


**Supplementary Figure 9 Hydrogen-bonding interactions between two chains in the asymmetric unit.**

**(a)** and **(b)** Hydrogen bond interactions in the Kae1-like domains. **(c)** Hydrogen bond interactions in the dimerization domains. The residues in different chains are shown as orange and blue stick models, and hydrogen-bonding interactions are indicated with black dashed lines.

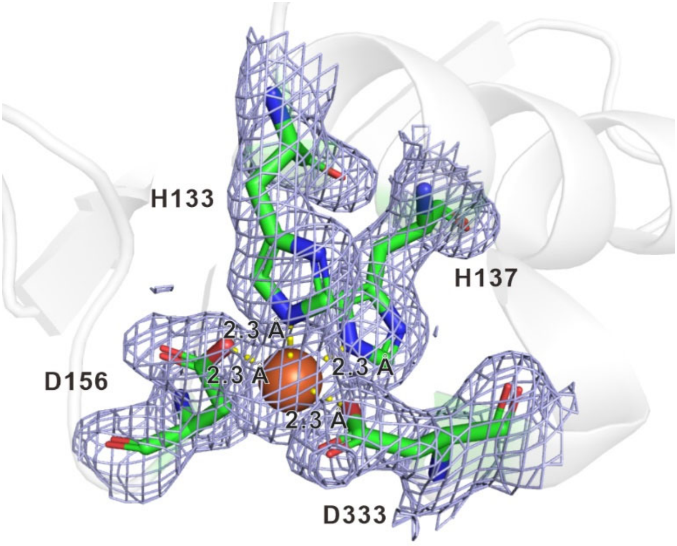


**Supplementary Figure 10 2*Fo-Fc* map for iron ion and its coordinating residues contoured at 1.0σ.**

Dash lines show the coordination of the metal with the distances labeled.

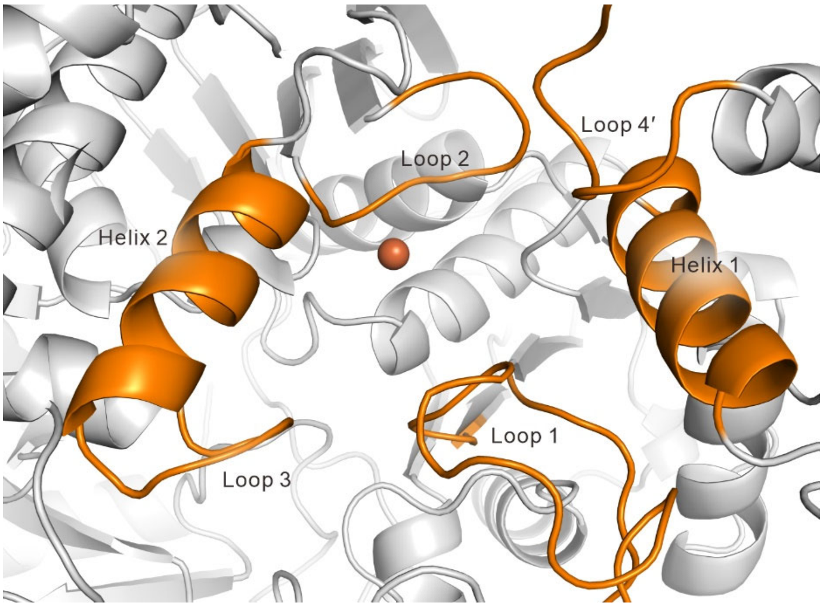


**Supplementary Figure 11 Substrate binding pocket of GdmN.**

Substrate binding pocket of GdmN is composed of two helixes and four loops: helix 1 (E85-E98), helix 2 (L186-L196), loop 1 (G8-A30), loop 2 (D156-H162), loop 3 (L197-D203) as well as loop 4′ (H238-L246 from the other monomer). Helix 1, helix 2, loop 1, loop 2, loop 3, and loop 4′ are colored in orange.

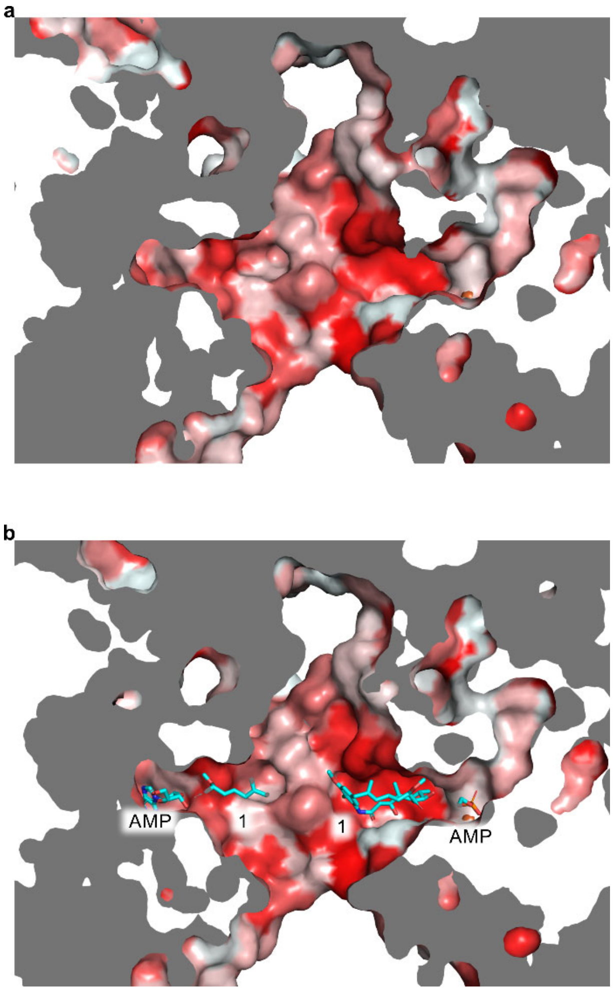


**Supplementary Figure 12 Clip view of catalytic chamber of GdmN formed by homodimer architecture.**

**(a)** Ligand-free catalytic chamber. **(b)** AMP and **1** in the catalytic chamber. The hydrophobic surface is colored in red, whereas the hydrophilic surface is colored in white. AMP and **1** are shown as cyan stick models.

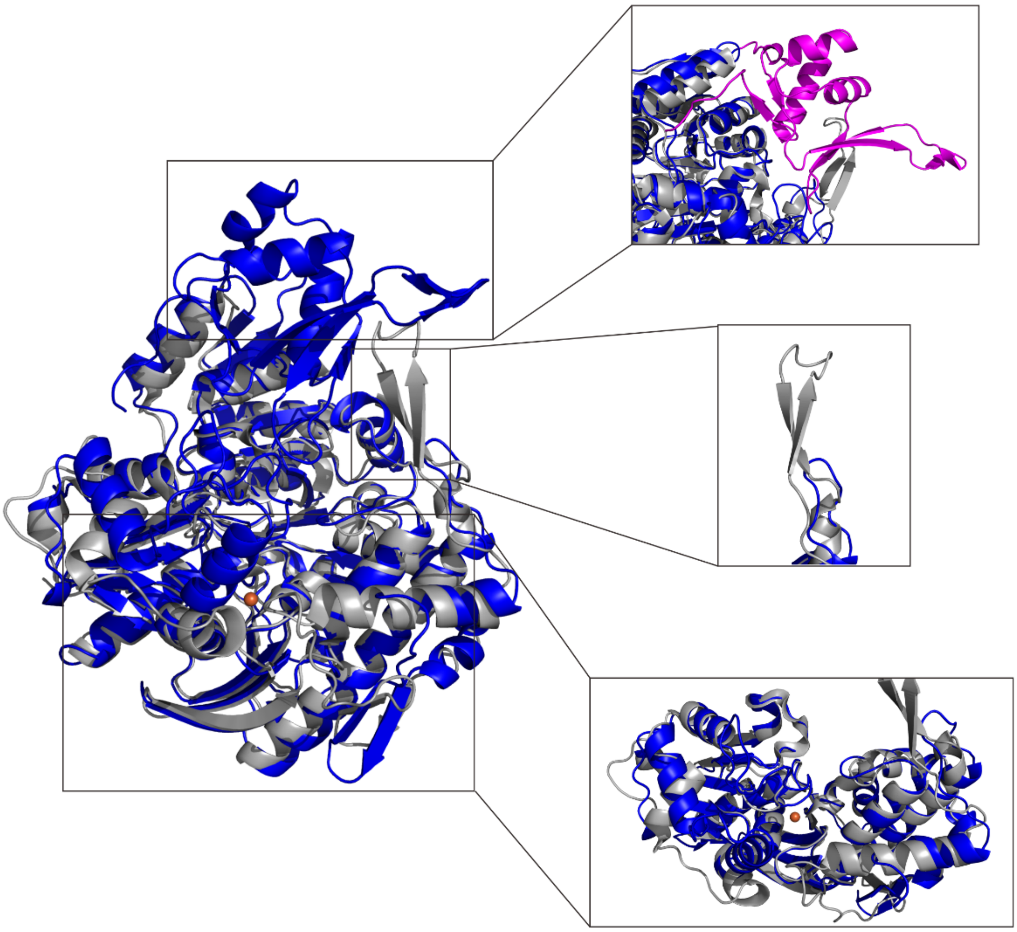

**Supplementary Figure 13 Superimposition of GdmN structure onto TobZ structure.**

The structure of GdmN is colored in blue, and the structure of TobZ is colored in grey. There are three differences between TobZ and GdmN, including the addition of dimerization domain (the domain shown in magenta in the upper picture), the loss of two-stranded β-sheet in the GdmN structure, as well as the broaden substrate binding pocket in Kae1-like domain of GdmN.

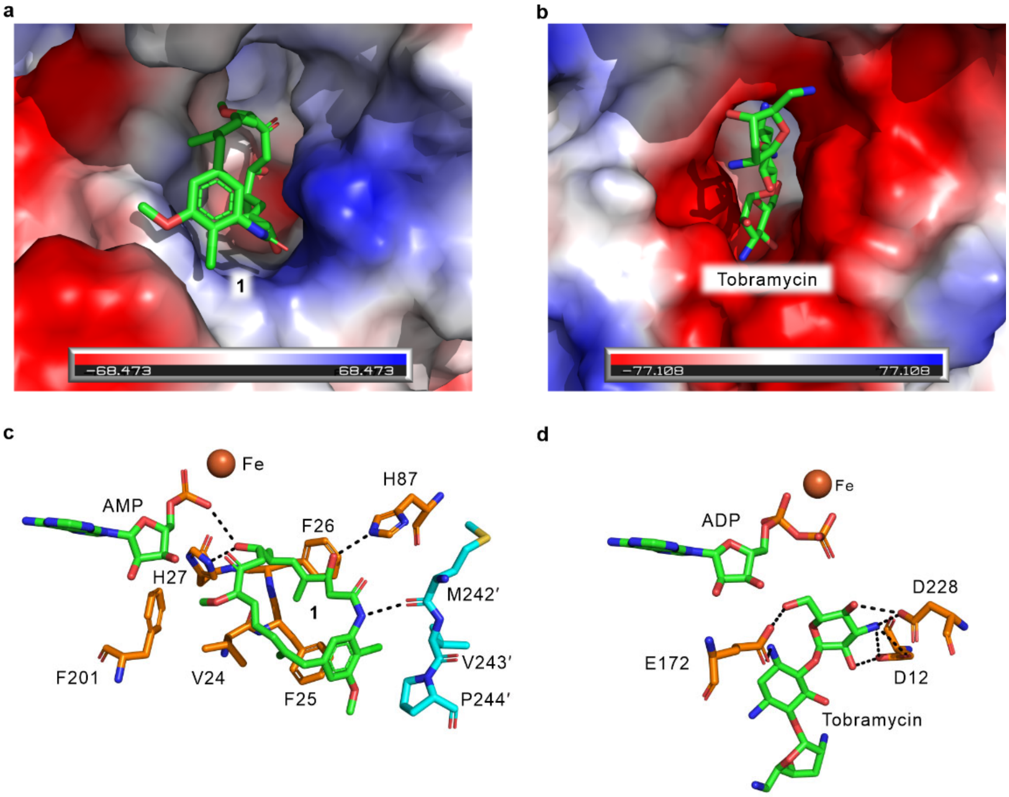


**Supplementary Figure 14 Electrostatic surface potential and interactions with ligands of GdmN and TobZ.**

**(a)** Electrostatic surface potential of GdmN. Electrostatics are colored from red (−68.473 *k*T/e) to blue (+68.473 *k*T/e). **(b)** Electrostatic surface potential of TobZ. Electrostatics are colored from red (−77.108 *k*T/e) to blue (+77.108 *k*T/e). **(c)** Interaction of **1** with surrounding residues in the GdmN/AMP/**1** structure. Hydrogen-bonding interactions are indicated with black dashed lines. AMP and **1** are shown as green stick models. The residues from this monomer are shown as orange stick models, and the residues from the partner monomer are shown as cyan stick models. **(d)** Interaction of tobramycin with surrounding residues in the TobZ complex structure. Hydrogen-bonding interactions are indicated with black dashed lines. Ligands are shown as green stick models, and the residues are shown as orange stick models.

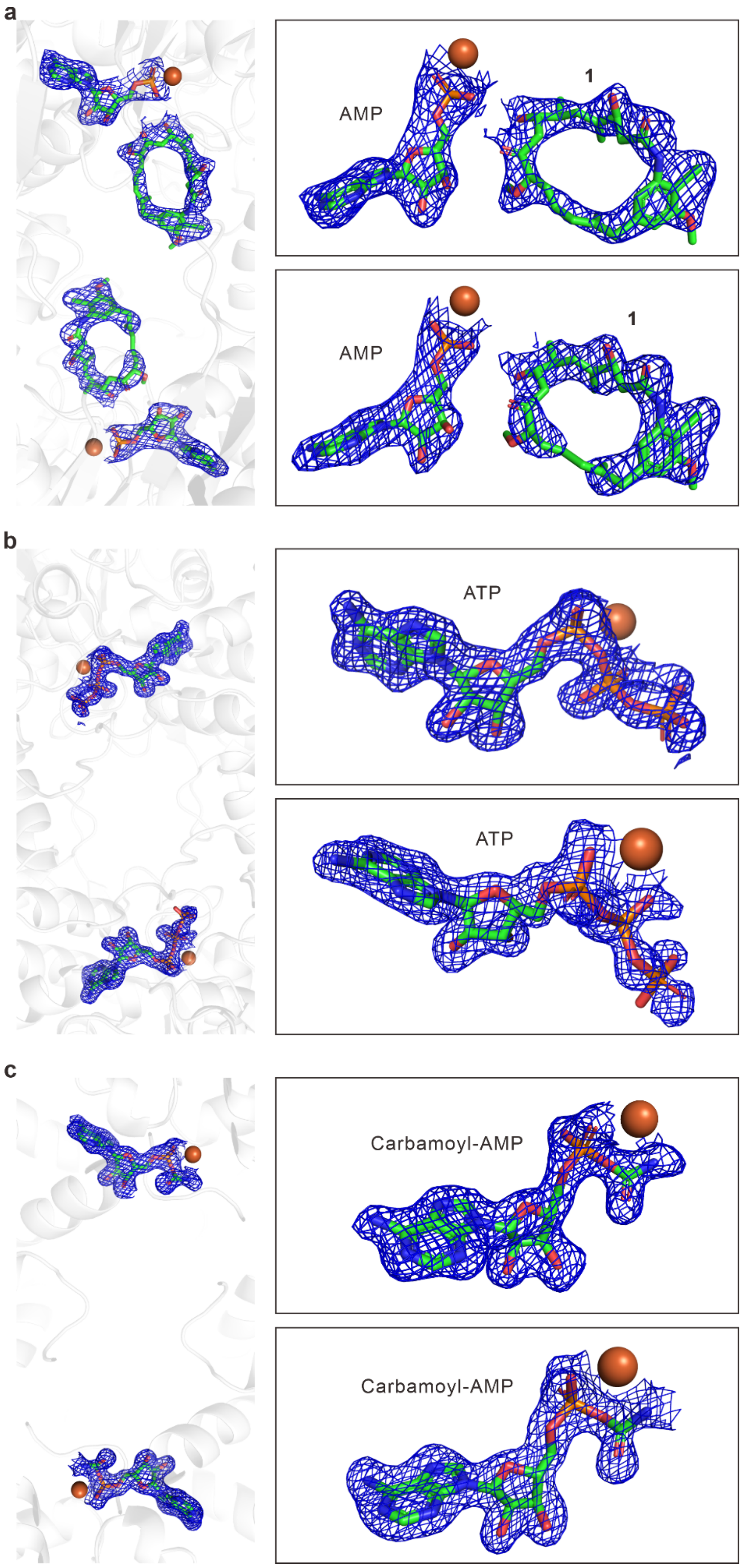

a
AMP
1
AMP
1
b
ATP
ATP
c
Carbamoyl-AMP
Carbamoyl-AMP

**Supplementary Figure 15 *Fo-Fc* omit maps for different ligands.**

**(a)** *Fo-Fc* omit maps contoured at 2.3 σ of AMP and **1** in the Kae1-like domain of the GdmN complexed with AMP and **1** (PDB: 7VZY). **(b)** *Fo-Fc* omit maps contoured at 2.5 σ of ATP in the Kae1-like domain of the GdmN complexed with ATP (PDB: 7VX0). **(c)** *Fo-Fc* omit maps contoured at 2.5 σ of carbamoyl-AMP in the Kae1-like domain of the GdmN complexed with carbamoyl-AMP (PDB: 7VYJ).

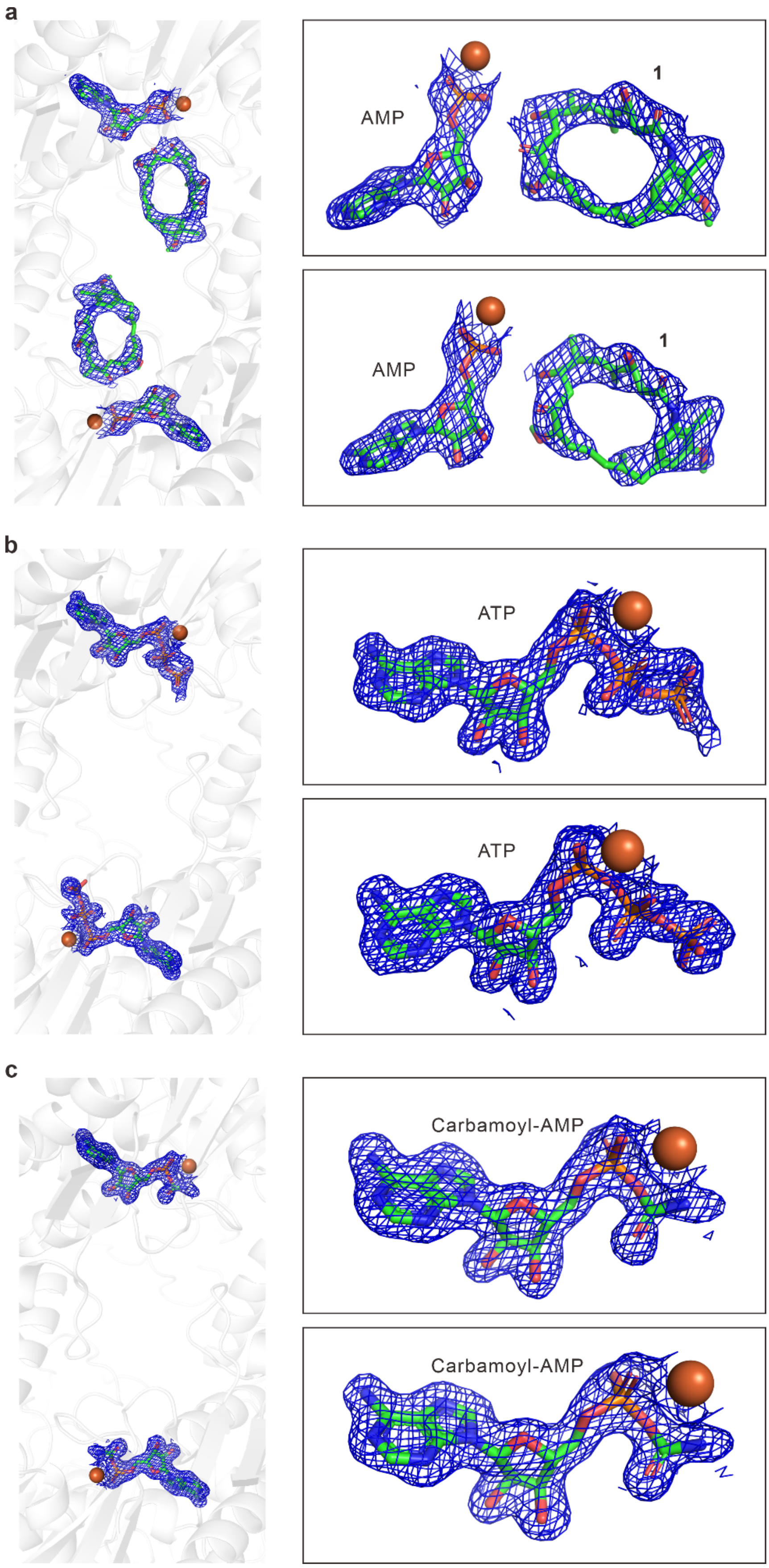
a
AMP
1
AMP
1
b
ATP
ATP
c
Carbamoyl-AMP
Carbamoyl-AMP

**Supplementary Figure 16 2*Fo-Fc* maps for different ligands contoured at 1.0σ.**

**(a)** Electron density of AMP and **1** in the Kae1-like domain of the GdmN complexed with AMP and **1** (PDB: 7VZY). **(b)** Electron density of ATP in the Kae1-like domain of the GdmN complexed with ATP (PDB: 7VX0). **(c)** Electron density of carbamoyl-AMP in the Kae1-like domain of the GdmN complexed with carbamoyl-AMP (PDB: 7VYJ).

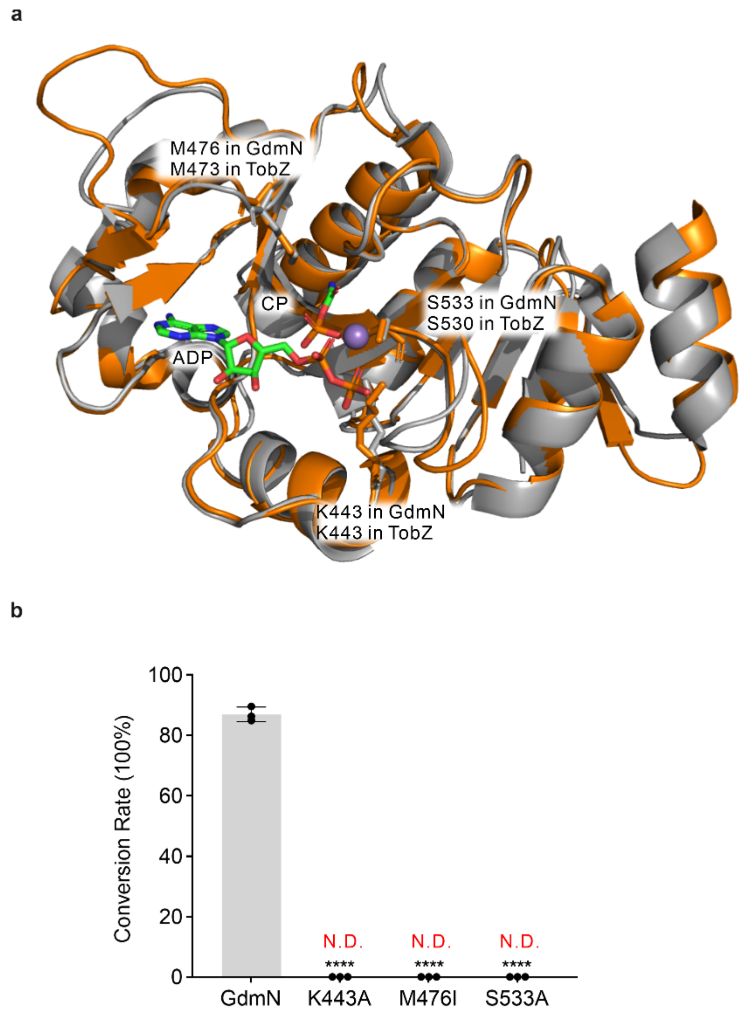


**Supplementary Figure 17 Analysis of the YrdC-like domain of GdmN with that of TobZ.**

**(a)** Superposition of the YrdC-like domain of GdmN with that of TobZ. The YrdC-like domain (G369-V567) of GdmN shares the same fold with the YrdC-like domain (D367-H570) of TobZ, with a 0.731 Å r.m.s.d. for 163 Cα atoms. The sequence alignment revealed that the active sites for adenylation of CP are highly conserved, with conservation of residues corresponding to K443/M476/S533 in GdmN and K443/M473/S530 in TobZ. **(b)** Catalytic activities of wild-type GdmN and its mutants. The activity assays of GdmN and mutants were performed with **1**, CP, ATP, and $MgCl_2$ at 30 °C for 12 h. N.D., products not detected. Wild-type GdmN was used as the control.

Graphs depict means ± SD (n = 3 independent experiments). Statistical analysis was performed by one-way ANOVA with Dunnett's multiple-comparison test (****$P < 0.0001$). Source data are provided as a Source Data file.

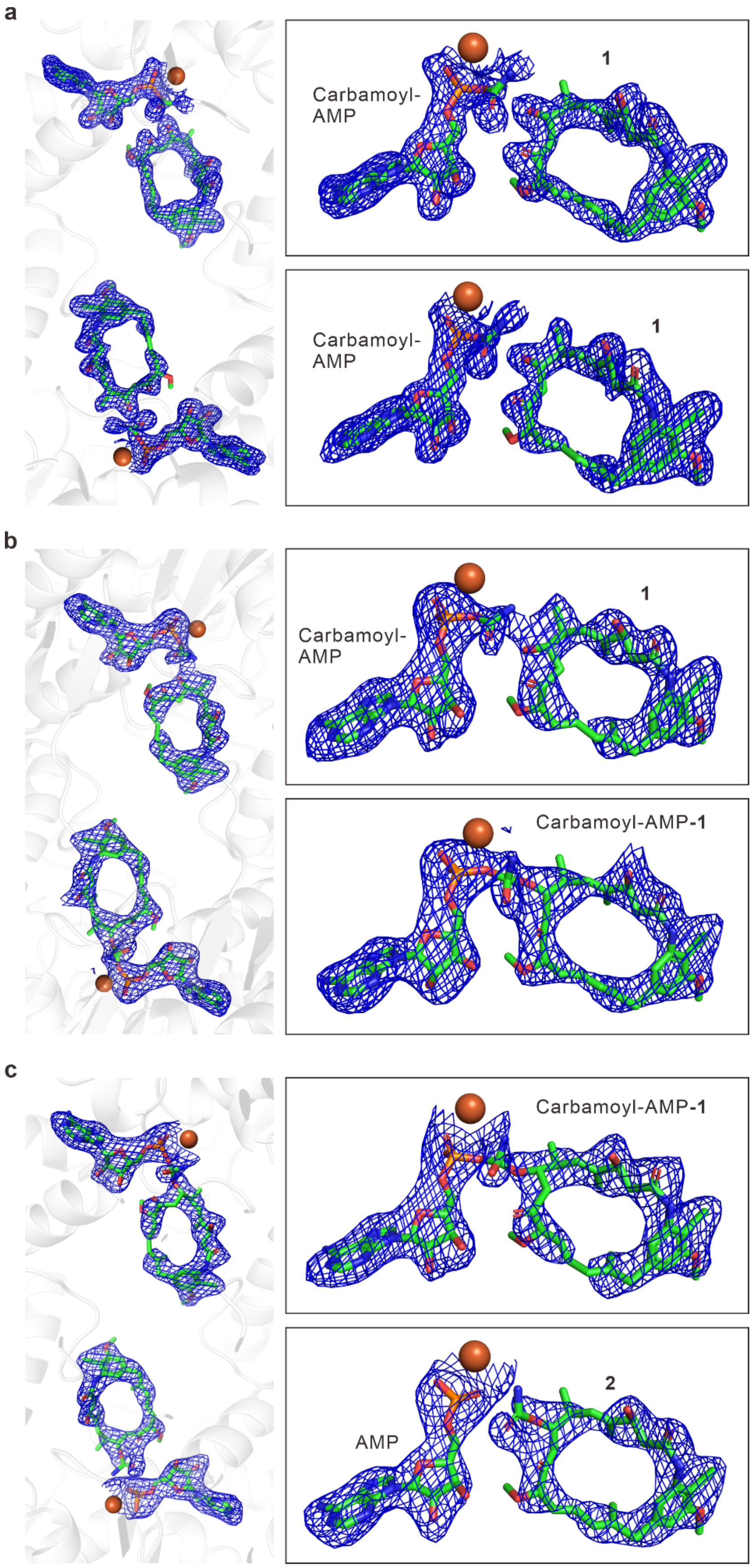
a
Carbamoyl-
AMP
1
Carbamoyl-
AMP
1
b
Carbamoyl-
AMP
1
Carbamoyl-AMP-1
c
Carbamoyl-AMP-1
AMP
2

**Supplementary Figure 18 *Fo-Fc* omit maps for different ligands.**

**(a)** *Fo-Fc* omit maps contoured at 2.3 σ of carbamoyl-AMP and **1** in the Kae1-like domain of the GdmN complexed with carbamoyl-AMP and **1** (PDB: 7VZN). **(b)** *Fo-Fc* omit maps contoured at 2.3 σ of carbamoyl-AMP-**1** natural tetrahedral intermediate, carbamoyl-AMP, and **1** in the Kae1-like domain of the GdmN complexed with a natural tetrahedral intermediate, carbamoyl-AMP, and **1** (PDB: 7VZZ). **(c)** *Fo-Fc* omit maps contoured at 2.3 σ of carbamoyl-AMP-**1** tetrahedral intermediate, **2**, and AMP in the Kae1-like domain of the GdmN complexed with a natural tetrahedral intermediate, AMP, and **2** (PDB: 7VYP).

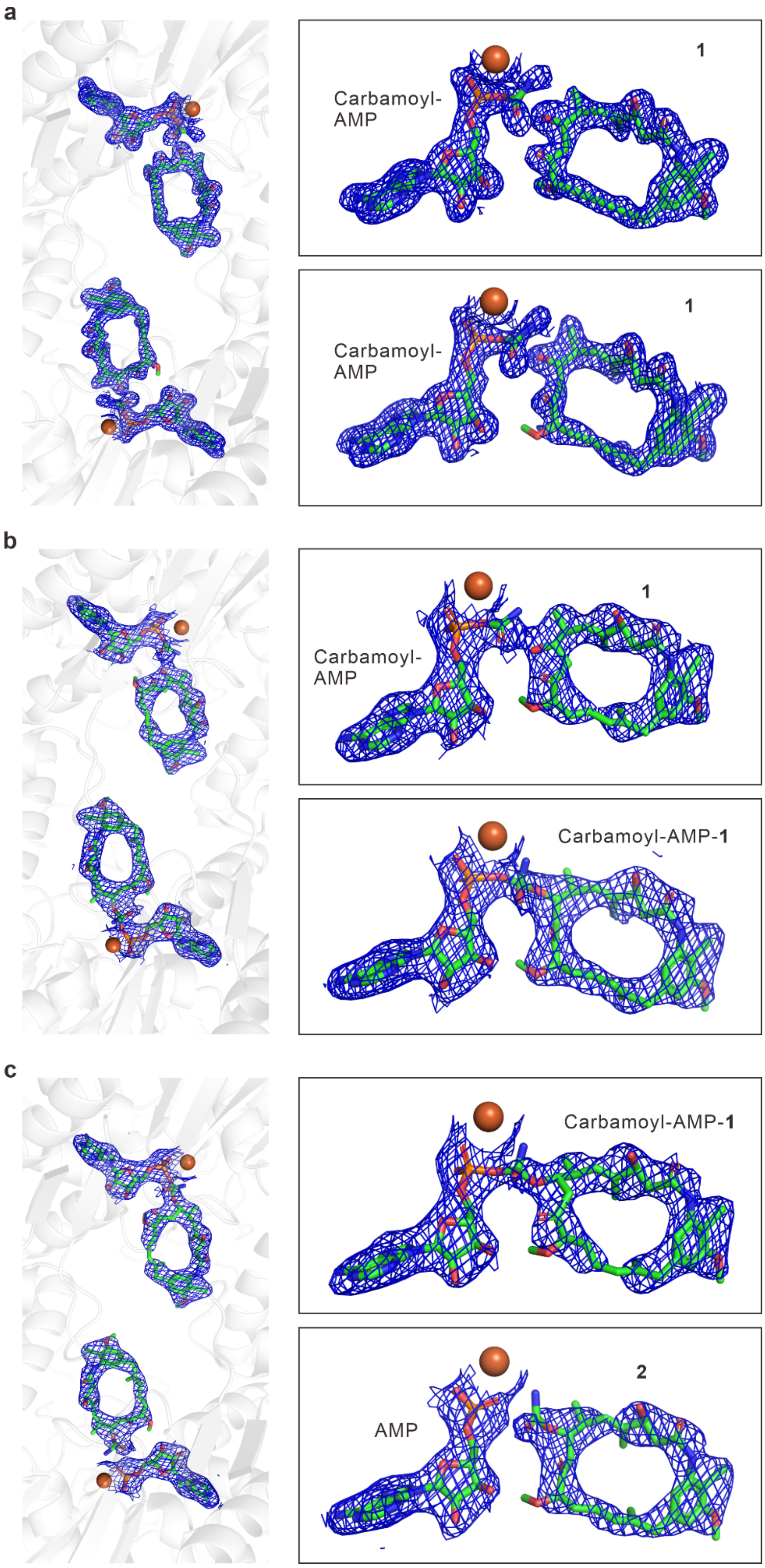
a
1
Carbamoyl-AMP
1
Carbamoyl-AMP
b
1
Carbamoyl-AMP
Carbamoyl-AMP-1
c
Carbamoyl-AMP-1
2
AMP

**Supplementary Figure 19 2*Fo-Fc* maps for different ligands contoured at 1.0σ.**

**(a)** Electron density of carbamoyl-AMP and **1** in the Kae1-like domain of the GdmN complexed with carbamoyl-AMP and **1** (PDB: 7VZN). **(b)** Electron density of carbamoyl-AMP, **1**, and carbamoyl-AMP-**1** natural tetrahedral intermediate in the Kae1-like domain of the GdmN complexed with carbamoyl-AMP, **1**, and a natural tetrahedral intermediate (PDB: 7VZZ). **(c)** Electron density of carbamoyl-AMP-**1** tetrahedral intermediate, **2**, and AMP in the Kae1-like domain of the GdmN complexed with a natural tetrahedral intermediate, AMP, and **2** (PDB: 7VYP).

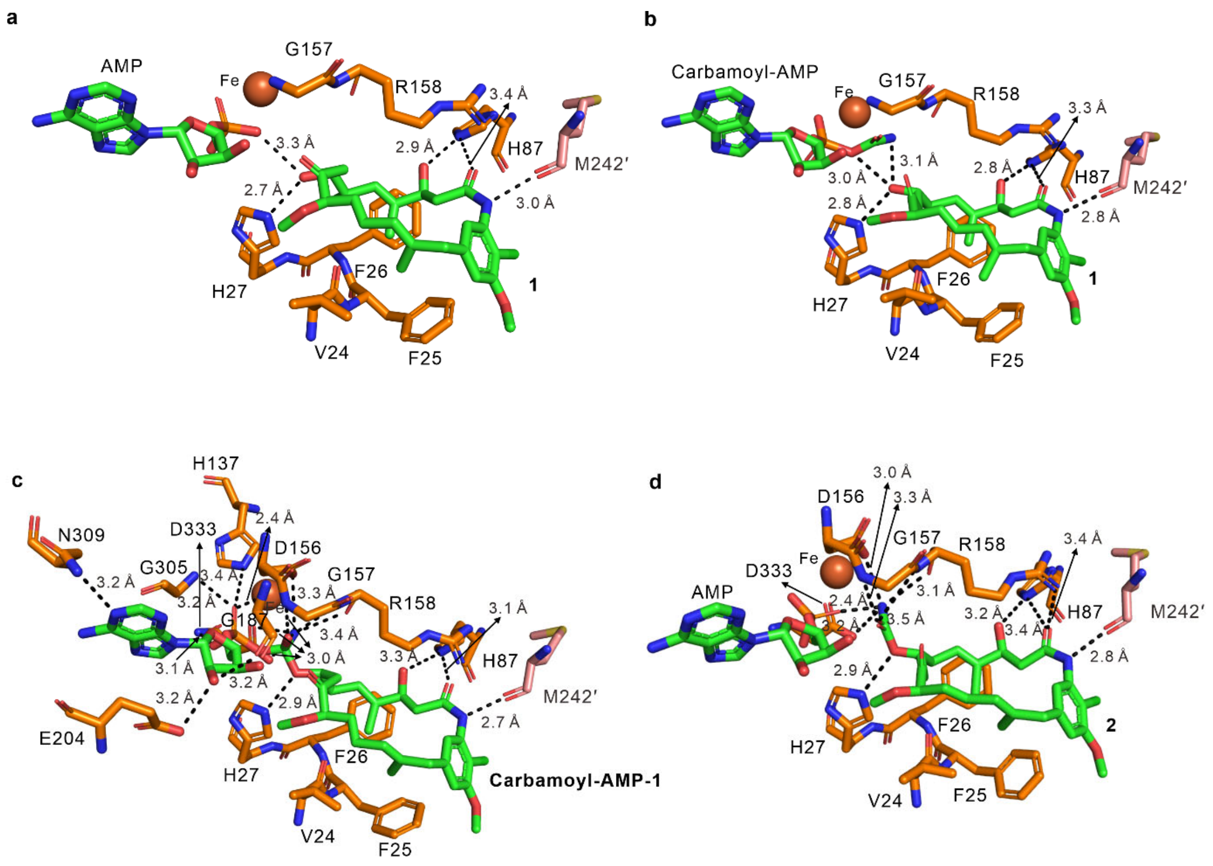


**Supplementary Figure 20 Interaction of ligands with surrounding residues in GdmN complexes structures.**

**(a)** Interactions of **1** with surrounding residues in the GdmN/AMP/**1** structure. **(b)** Interaction of **1** with surrounding residues in the GdmN/carbamoyl-AMP/**1** structure. **(c)** Interaction of carbamoyl-AMP-**1** natural tetrahedral intermediate with surrounding residues in the GdmN/carbamoyl-AMP-**1** structure. **(d)** Interaction of **2** with surrounding residues in the GdmN/AMP/**2** structure. The residues in this chain are shown as orange stick models, while M242′ from the partner monomer is shown as pink. Hydrogen-bonding interactions are indicated with black dashed lines.

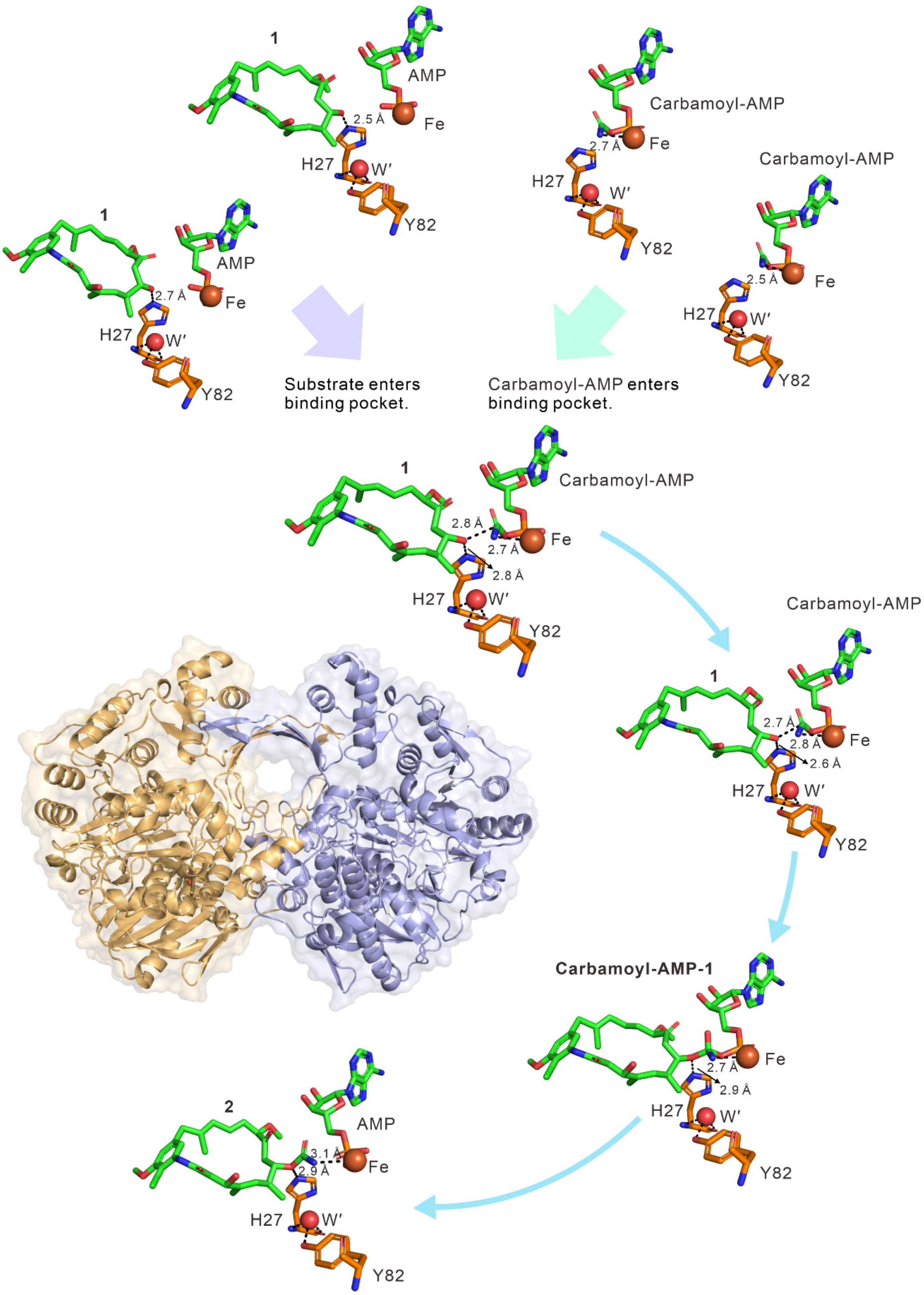

1
AMP
2.5 Å
Fe
H27
W′
Y82
Carbamoyl-AMP
2.7 Å
Fe
H27
W′
Y82
Carbamoyl-AMP
2.5 Å
Fe
H27
W′
Y82
1
AMP
2.7 Å
Fe
H27
W′
Y82
Substrate enters binding pocket.
Carbamoyl-AMP enters binding pocket.
1
Carbamoyl-AMP
2.8 Å
2.7 Å
2.8 Å
Fe
H27
W′
Y82
Carbamoyl-AMP
1
2.7 Å
2.8 Å
2.6 Å
Fe
H27
W′
Y82
Carbamoyl-AMP-1
Fe
2.7 Å
2.9 Å
H27
W′
Y82
2
AMP
3.1 Å
2.9 Å
Fe
H27
W′
Y82

**Supplementary Figure 21 Crystallographic snapshots during carbamoylation process.**

The co-crystal structures of GdmN/carbamoyl-AMP, GdmN/AMP/**1** and GdmN in complex with different intermediates showed the distance between different ligands and the active site H27, depicting different stages during carbamoylation process. In all experimental data, a water molecule (W′) interacting with the hydroxy group of Y82 appears near H27, forming hydrogen-bonding interactions with the backbone amide and carbonyl of H27. Green stick model, structures of ligands; orange stick model, structures of H27 and Y82; red sphere, W′. Key distances are given in Å.

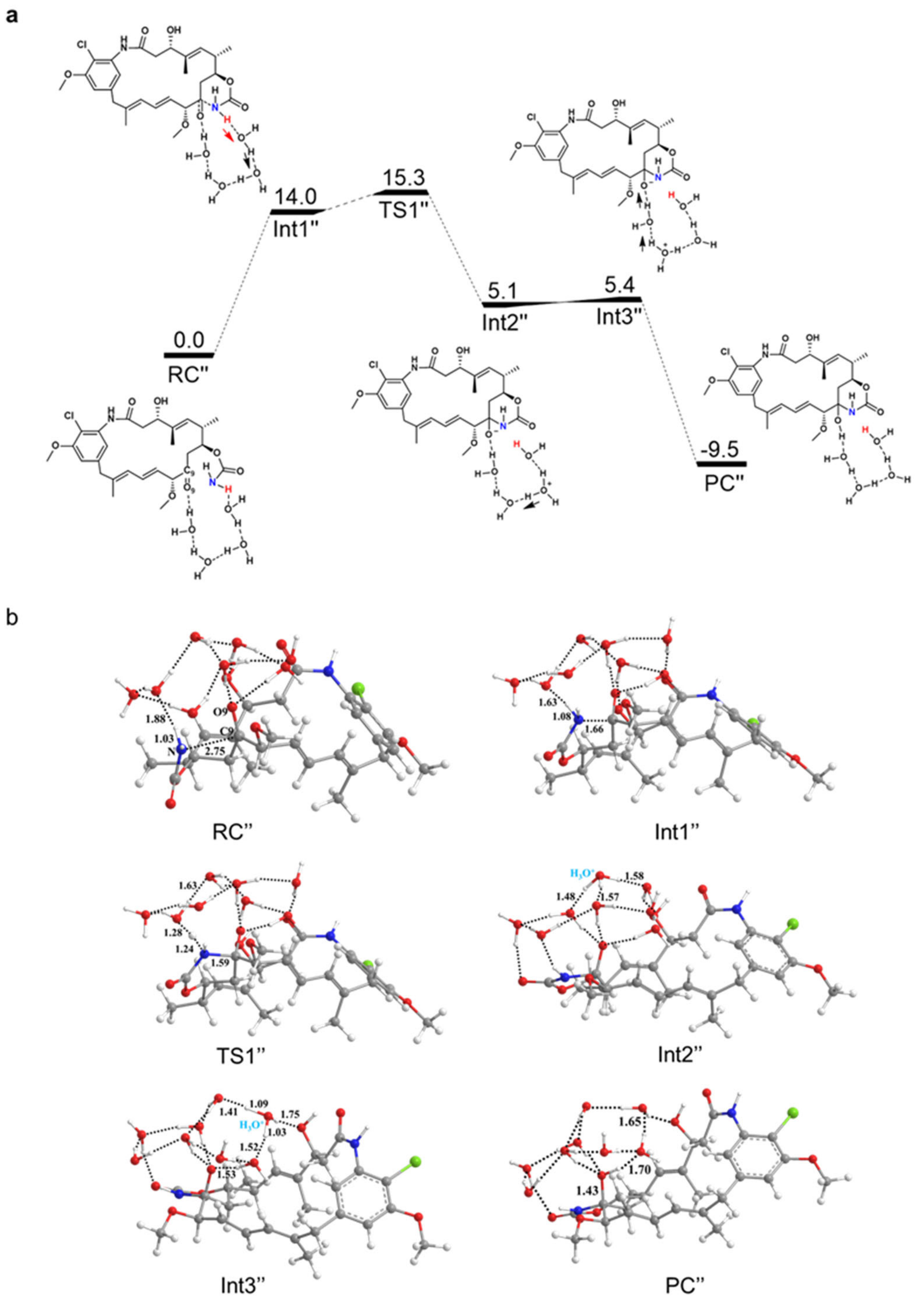


**Supplementary Figure 22 The QM calculation results for the conversion from 2 to 3.**

**(a)** Cluster-continuum model calculated free energy profile (kcal/mol) for the conversion from **2** to **3** with 8 explicit water molecules at the BMK/6-311++G(d, p)

level. **(b)** Optimized geometry structures in the reaction at the B3LYP/6-31G(d) level. Key distances are given in Å.

Using cluster-continuum model calculations, we have re-evaluated the reaction barrier for the conversion from **2** to **3**. The reaction is initiated by the approach of the amino N atom to the C9 atom of carbonyl group leading to the metastable Int1″ (see the scanned energy profile in Supplementary Figure 23). Next, one proton from the amino group transfers to a nearby water via TS1″, leading to the hydrated $H_3O^+$ core that is stabilized by the surrounding strong H-bonding interactions (see Supplementary Figure 22b). This step involves an overall barrier of 15.3 kcal/mol (RC″→Int2″). The following successive proton transfer from the $H_3O^+$ core to the O9 atom of the initial carbonyl group (Int2″→Int3″→PC″) can afford the final product **3** (PC″). Thus. our calculations show that the conversion from **2** to **3** is relatively facile in water solution.

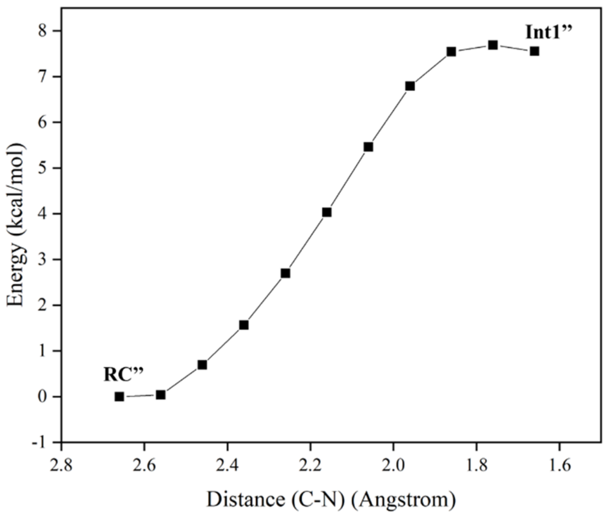


**Supplementary Figure 23 B3LYP/6-31G(d)-scanned energy profile (in kcal/mol) for the approach of the amino N atom to the C9 atom of the carbonyl group.**

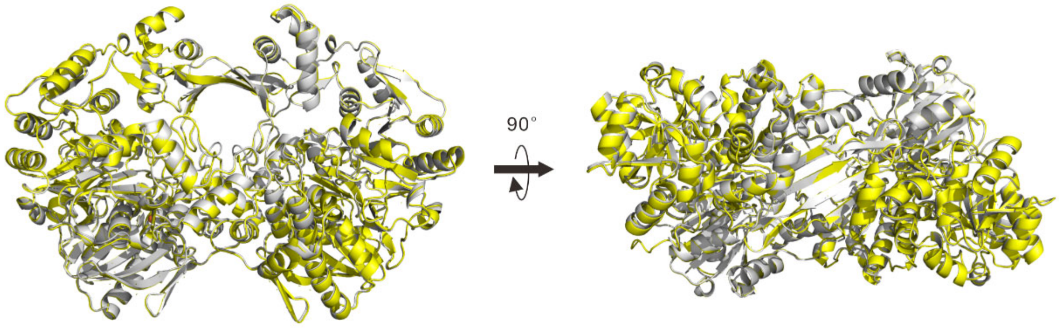


**Supplementary Figure 24 Superimposition of the Y82F structure (yellow) onto the GdmN/AMP/ 1 structure (grey) with an r.m.s.d. value of 0.38 Å.**

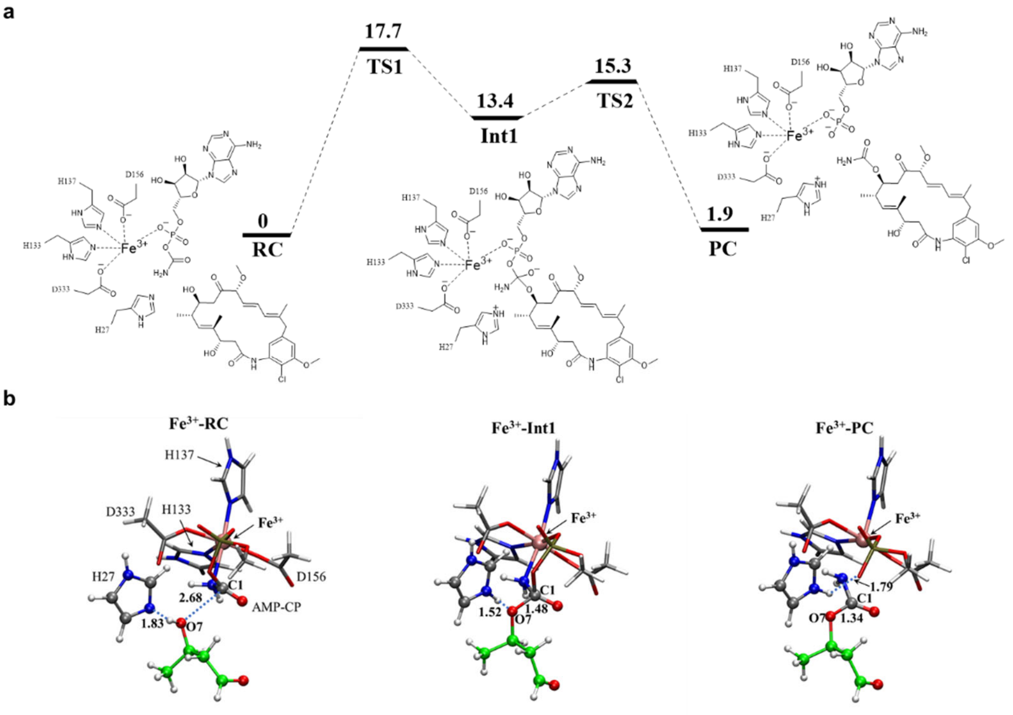


**Supplementary Figure 25 The QM/MM calculated mechanisms for the enzymatic reactions catalyzed by GdmN.**

**(a)** QM(UB3LYP/B2)/MM relative energies (kcal/mol) for the enzymatic reaction from **1** to **2** for the Fe(III). The dispersion corrections are included in the relative energies. **(b)** QM(UB3LYP/B1)/MM-optimized geometries of key species involved in the reaction. Key distances are given in Å. The green colored structure is the truncated structure of the substrate **1**. For clarity, the protein scaffold was omitted.

The enzymatic reaction from **1** to **2** was studied by QM/MM calculations, while the non-enzymatic reaction from the **2** to **3** occurring in water was studied by the QM-based cluster-continuum model calculations (Supplementary Figure 22). For the Fe(III) state, three spin states: doublet, quartet and sextet have been calculated. We found that the ground state of RC corresponds to the sextet state, while the quartet state and the doublet state is 13.0 kcal/mol and 15.6 kcal/mol higher than the sextet state, respectively.

As such, all QM/MM calculations are limited to the sextet state (Supplementary Figure 25a). As shown in Supplementary Figure 25a, the reaction is initiated by the proton transfer from the hydroxyl group of C7 to His27, which is coupled to the nucleophilic attack of the hydroxyl O7 to C1 of the carbamoyl-AMP, leading to the formation of the tetrahedral intermediate (Int1). The reaction encounters a barrier of 17.7 kcal/mol (RC →Int1). Then, the decomposition of the tetrahedral intermediate leads to the formation of the product **2**, which involves a tiny barrier of 1.9 kcal/mol (Int1→PC). Thus, the rate-limiting step is the formation of the tetrahedral intermediate, rather than the decomposition of tetrahedral intermediate as proposed before.

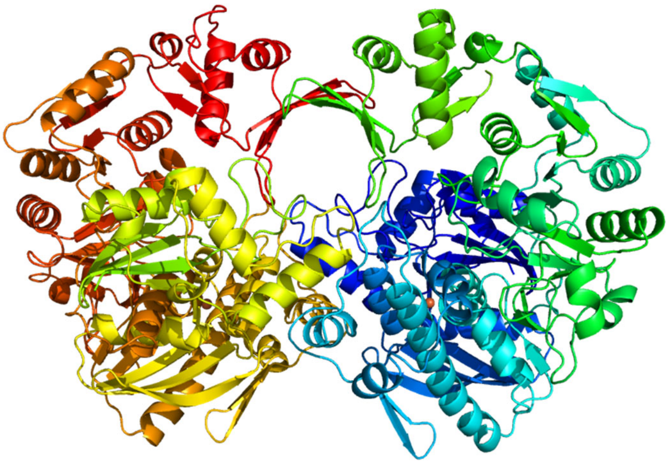

**Supplementary Figure 26 The overall structure model of Asc21b constructed by SWISS-MODEL server.**

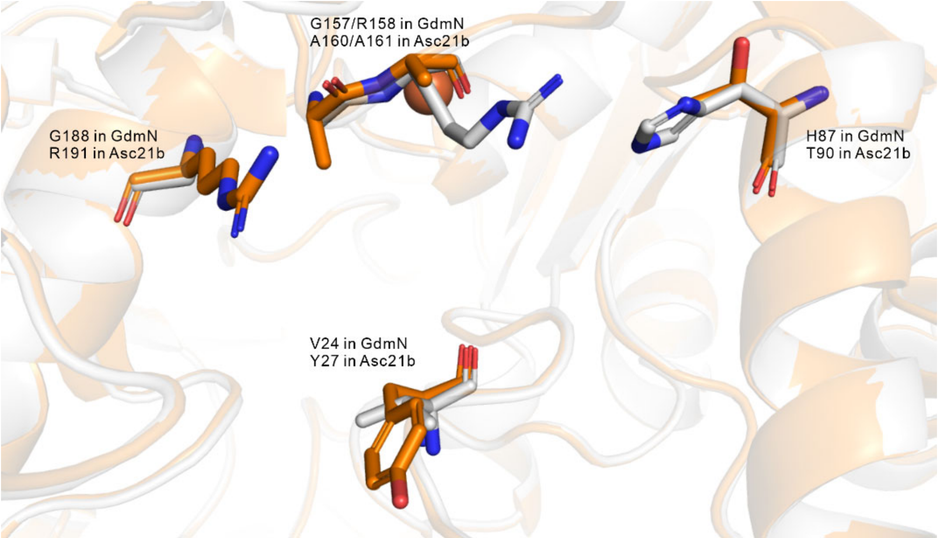


**Supplementary Figure 27 Proposed residues involved in substrate orientation.**

Analysis of GdmN (grey) and Asc21b (orange) structural elements revealed that five residues might influence substrate orientation. The residues in Asc21b are shown as orange stick models, and the residues in GdmN are shown as grey stick models.

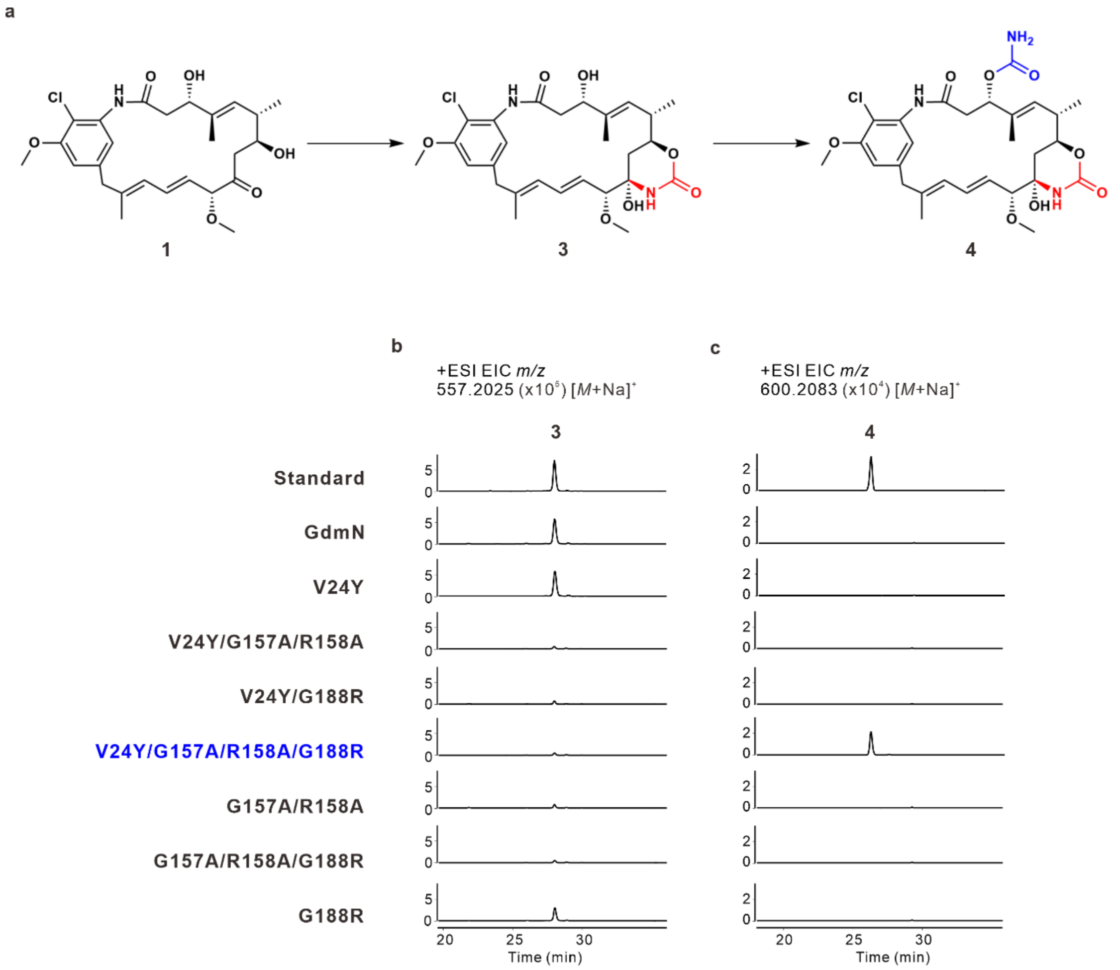


**Supplementary Figure 28 The LC-MS analysis of GdmN mutants towards 1 and 3.**

**(a)** The di-*O*-carbamoylation reaction. **(b)** The extracted ion chromatograms (EIC) of **3** (theoretical *m/z* 557.2025 $[M+\text{Na}]^+$) in GdmN mutants catalyzed reaction towards **1**. **(c)** The extracted ion chromatograms (EIC) of **4** (theoretical *m/z* 600.2083 $[M+\text{Na}]^+$) in GdmN mutants catalyzed reaction towards **3**. The activity assays of the mutants were performed with **1** or **3**, CP, ATP, and $MgCl_2$ at 30 °C for 12 h.

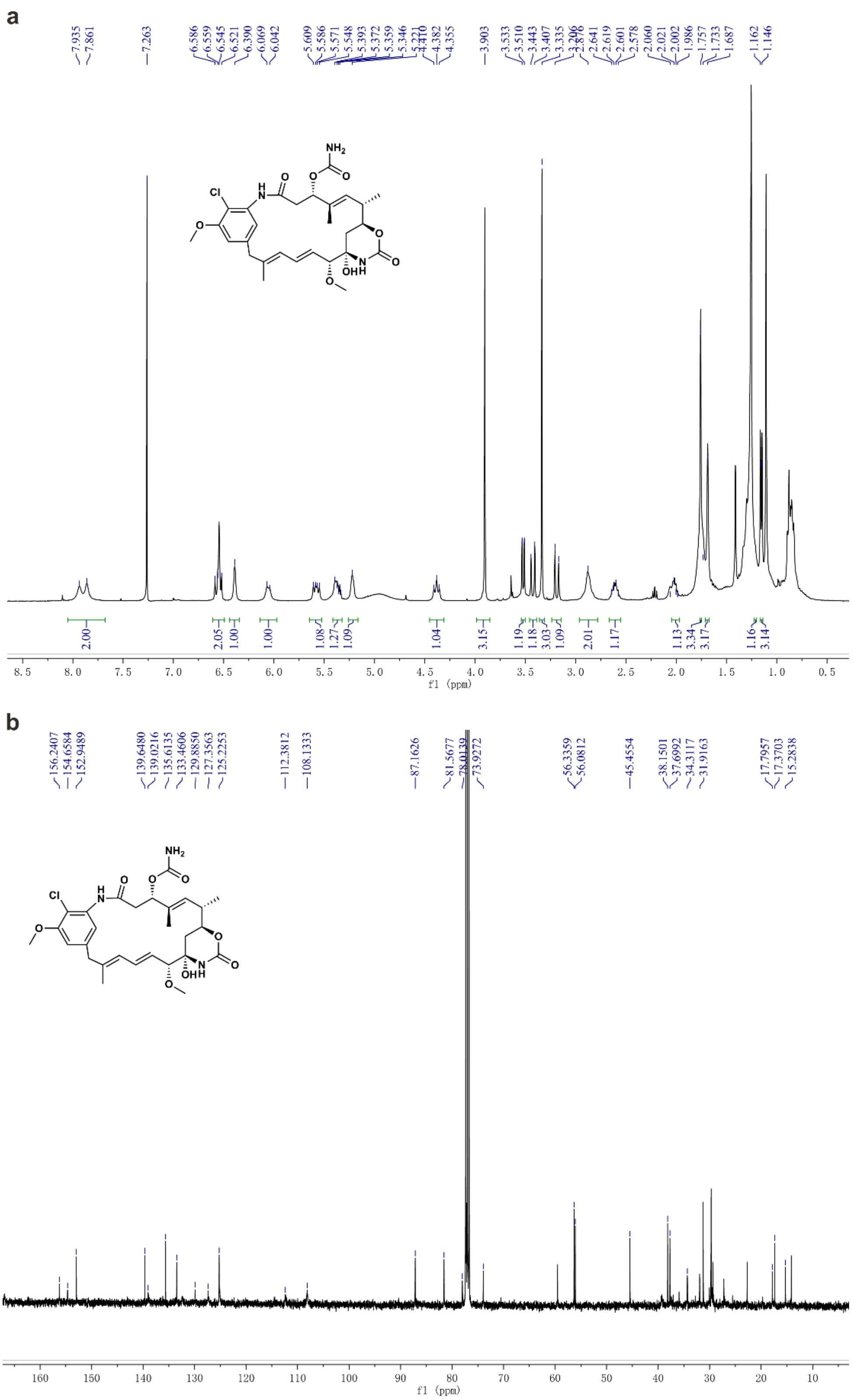


**Supplementary Figure 29 $^{1}$H NMR spectrum (a) and $^{13}$C NMR spectrum (b) of 4 in $CDCl_3$.**

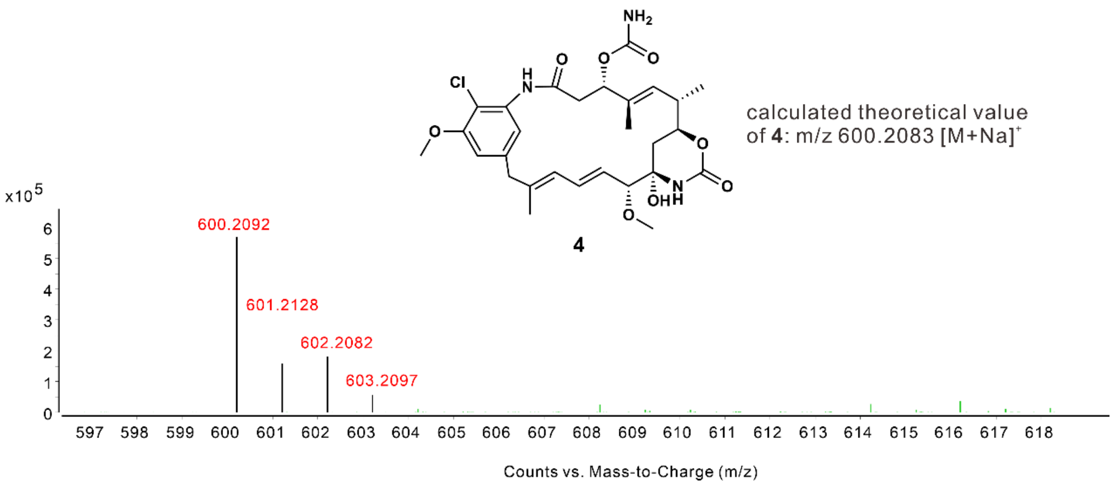


**Supplementary Figure 30 HR-ESI-MS analysis of 4.**

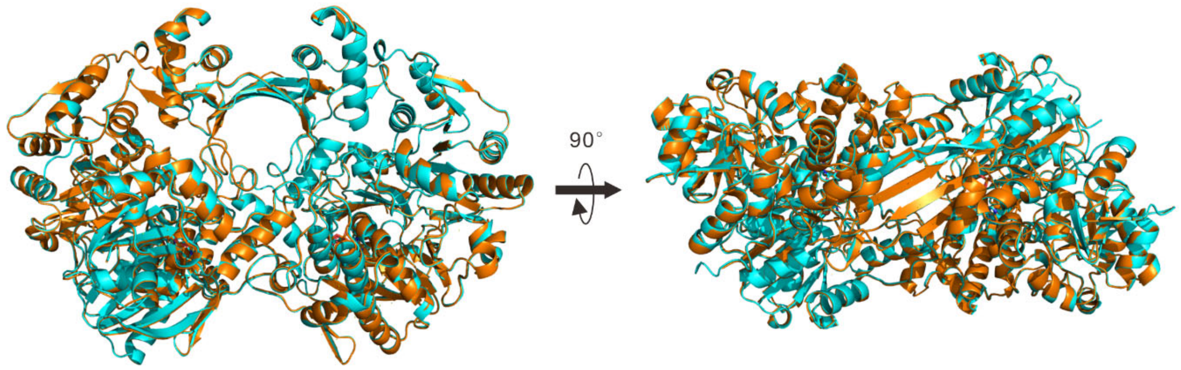


**Supplementary Figure 31 Superimposition of the GdmN V24Y/G157A/R158A/G188R mutant complexed with carbamoyl-AMP (orange) onto the GdmN/carbamoyl-AMP/1 complex (cyan).**

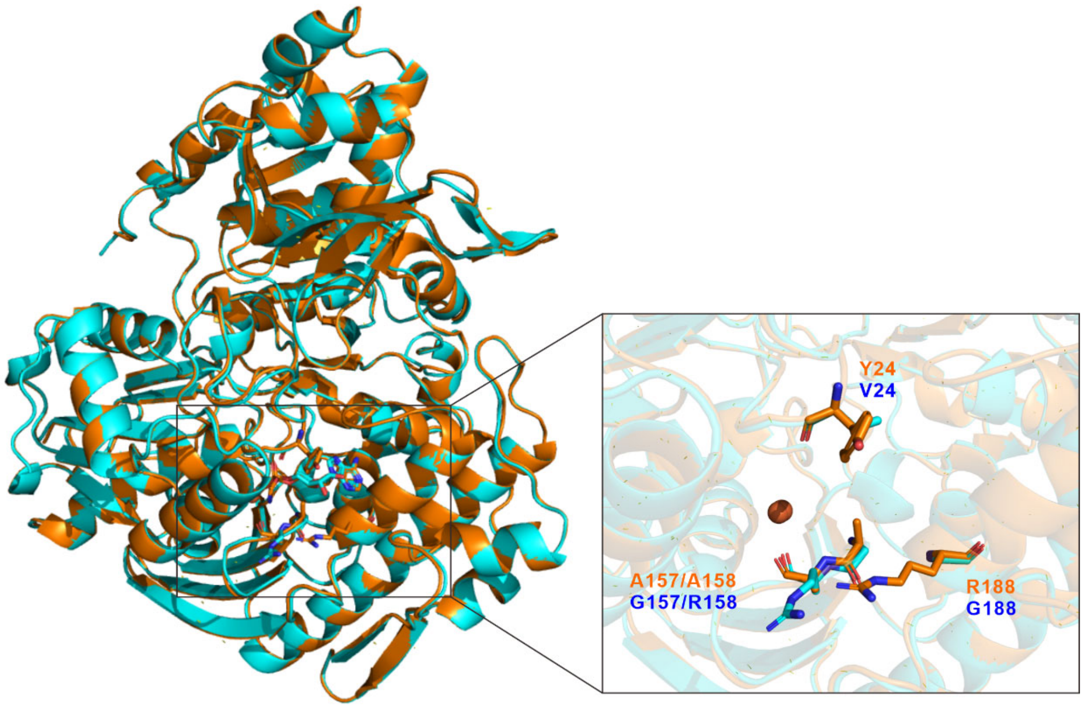


**Supplementary Figure 32 Structural analysis of the GdmN V24Y/G157A/R158A/G188R mutant and GdmN.**

The structure of the GdmN V24Y/G157A/R158A/G188R mutant is colored in orange, and the GdmN structure is colored in cyan.

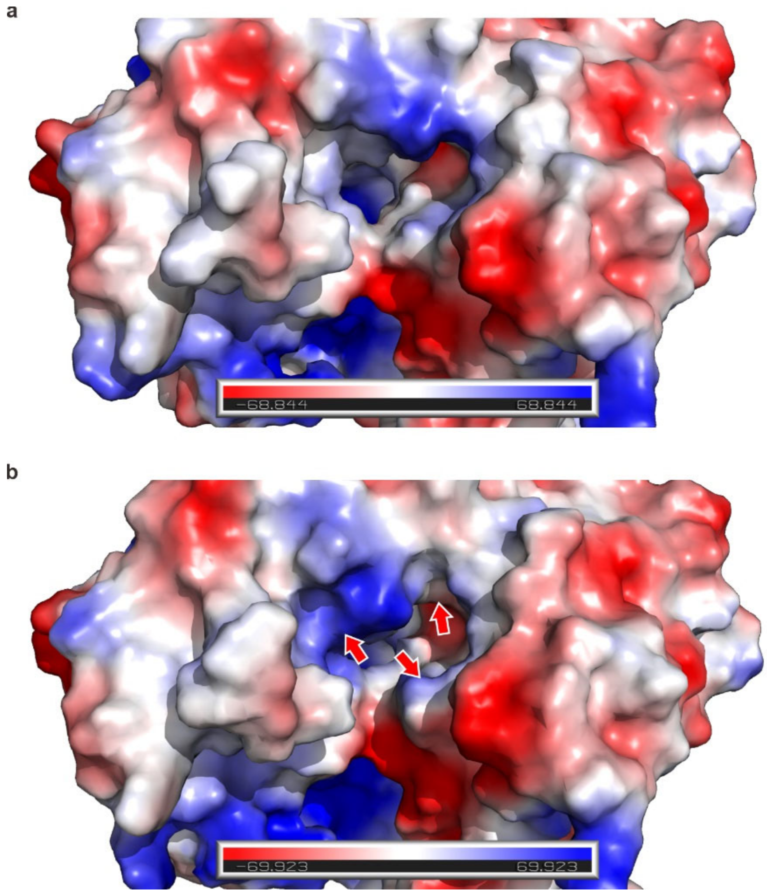


**Supplementary Figure 33 Electrostatic surface potential of GdmN and the GdmN V24Y/G157A/R158A/G188R mutant.**

**(a)** Electrostatic surface potential of GdmN. Electrostatics are colored from red (−68.844 *k*T/e) to blue (+68.844 *k*T/e). **(b)** Electrostatic surface potential of the GdmN V24Y/G157A/R158A/G188R mutant. Electrostatics are colored from red (−69.923 *k*T/e) to blue (+69.923 *k*T/e). The differences are marked with red narrows.

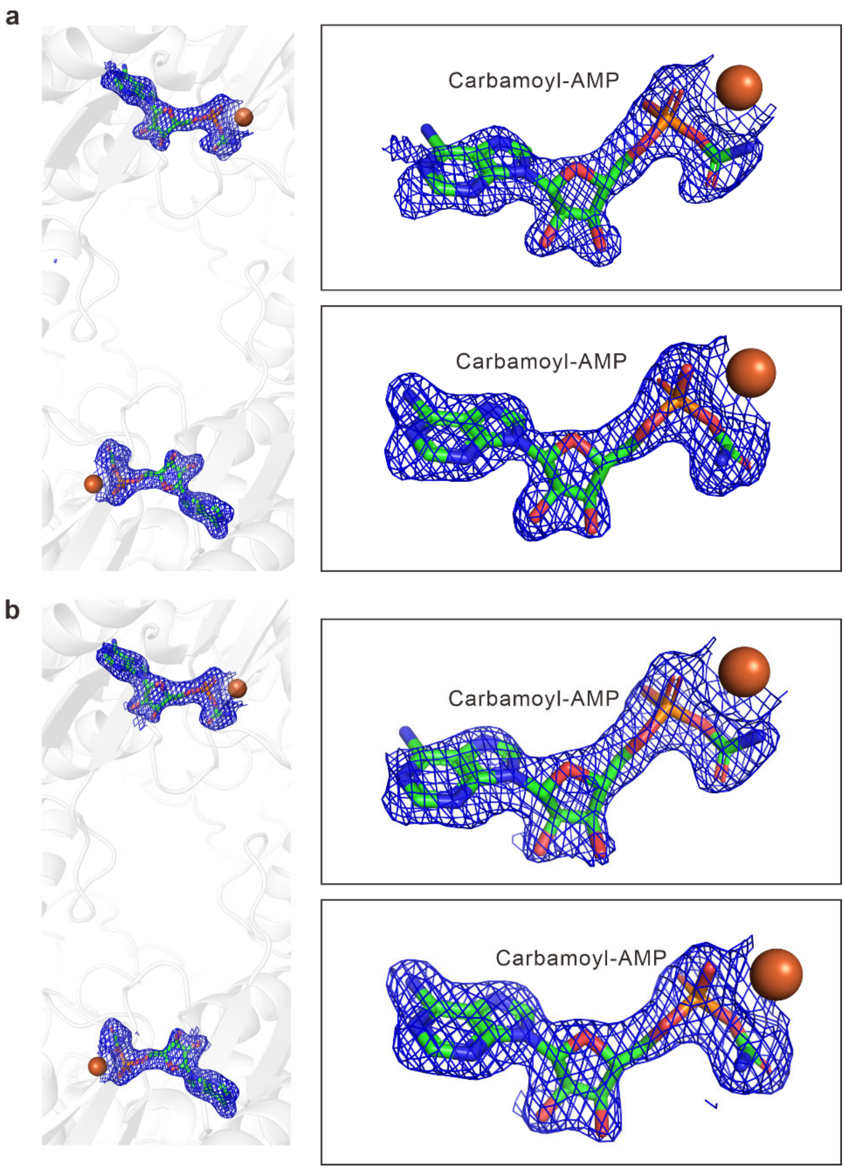


**Supplementary Figure 34 *Fo-Fc* omit maps and 2*Fo-Fc* maps of carbamoyl-AMP in the Kae1-like domain of the GdmN V24Y/G157A/R158A/G188R mutant in complex with carbamoyl-AMP (PDB: 7VZQ).**

**(a)** *Fo-Fc* omit maps contoured at 2.5 σ of carbamoyl-AMP in the Kae1-like domain of the GdmN V24Y/G157A/R158A/G188R mutant in complex with carbamoyl-AMP. **(b)** *2Fo-Fc* maps contoured at 1.0 σ of carbamoyl-AMP in the Kae1-like domain of the GdmN V24Y/G157A/R158A/G188R mutant in complex with carbamoyl-AMP.

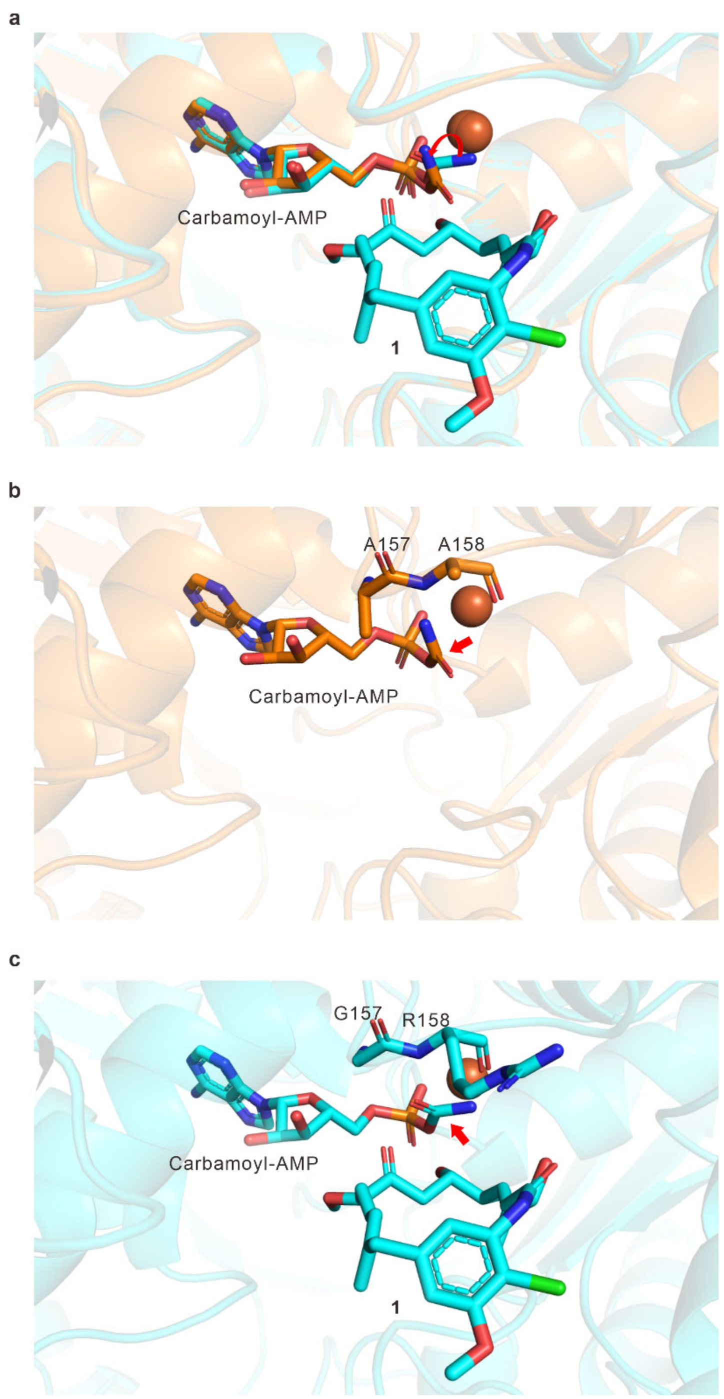


**Supplementary Figure 35 Binding position of carbamoyl-AMP in the GdmN V24Y/G157A/R158A/G188R mutant complexed with carbamoyl-AMP and the GdmN/carbamoyl-AMP/1 complex.**

**(a)** Superimposition of the GdmN V24Y/G157A/R158A/G188R mutant complexed with carbamoyl-AMP (orange) onto the GdmN/carbamoyl-AMP/**1** complex (cyan). **(b)**

Binding position of carbamoyl-AMP in the GdmN V24Y/G157A/R158A/G188R mutant complexed with carbamoyl-AMP (orange). The residues and carbamoyl-AMP are shown as orange stick models. **(c)** Binding position of carbamoyl-AMP in the GdmN/carbamoyl-AMP/**1** complex (cyan). The residues, carbamoyl-AMP, and **1** are shown as cyan stick models. The carbamoyl groups are marked with red narrows.

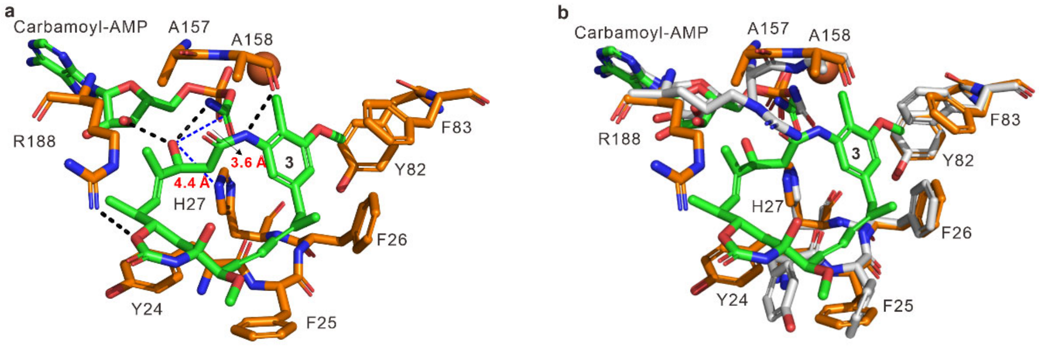


**Supplementary Figure 36 Computational docking of 3 into the GdmN V24Y/G157A/R158A/G188R mutant structure.**

**(a)** Computational docking of **3** into the mutant structure. **3** bound to the mutant in a direction in which the C-3 oxygen is placed close to carbamoyl group of carbamoyl-AMP and the side chain of H27 at the distance of 3.6 and 4.4 Å, respectively. Hydrogen-bonding interactions are indicated with black dashed lines. **3** and carbamoyl-AMP are shown as green stick models, and the residues are shown as orange stick models. **(b)** Superimposition of computational analysis results into the mutant/carbamoyl-AMP complex structure. **3** and carbamoyl-AMP are shown as green stick models. The residues in computational analysis results are shown as orange stick models, whereas the residues in the mutant/ carbamoyl-AMP structure are shown as grey stick models.

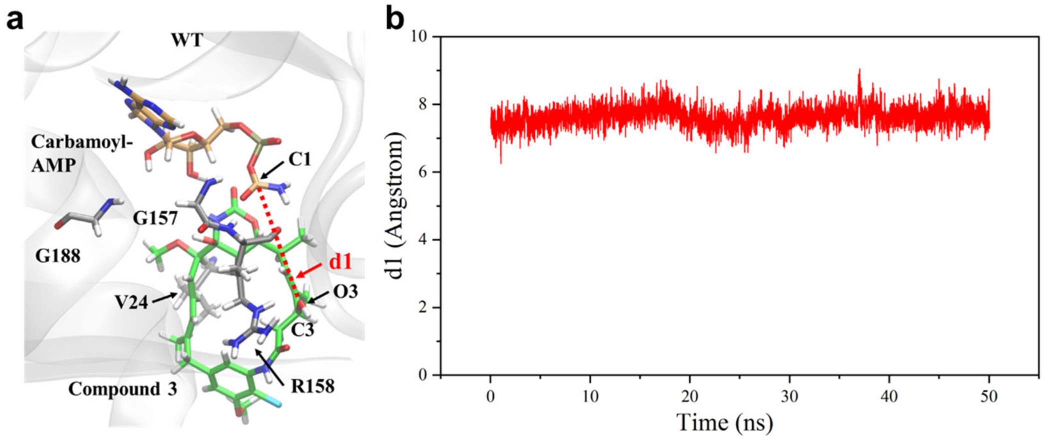


**Supplementary Figure 37 The MD simulations of the wild-type GdmN with 3.**

**(a)** Representative structure of the wild-type GdmN with **3** from the MD simulations.

**(b)** The fluctuation of the distance between the O3 atom of **3** and the C1 atom of carbamoyl-AMP (denoted as d1).

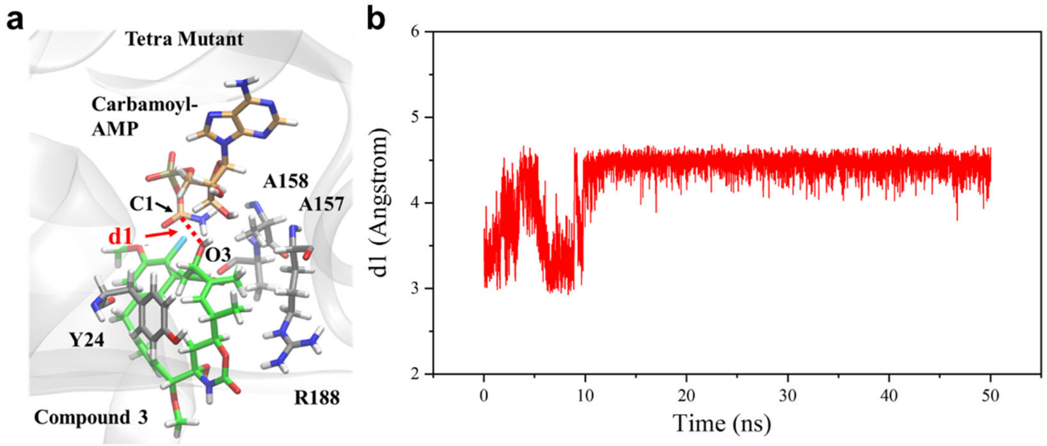


**Supplementary Figure 38 The MD simulations of the tetra-mutant GdmN with 3.**

**(a)** Representative structure of the tetra-mutant GdmN with **3** from the MD simulations.

**(b)** The fluctuation of the distance between the O3 atom of **3** and the C1 atom of carbamoyl-AMP (denoted as d1).

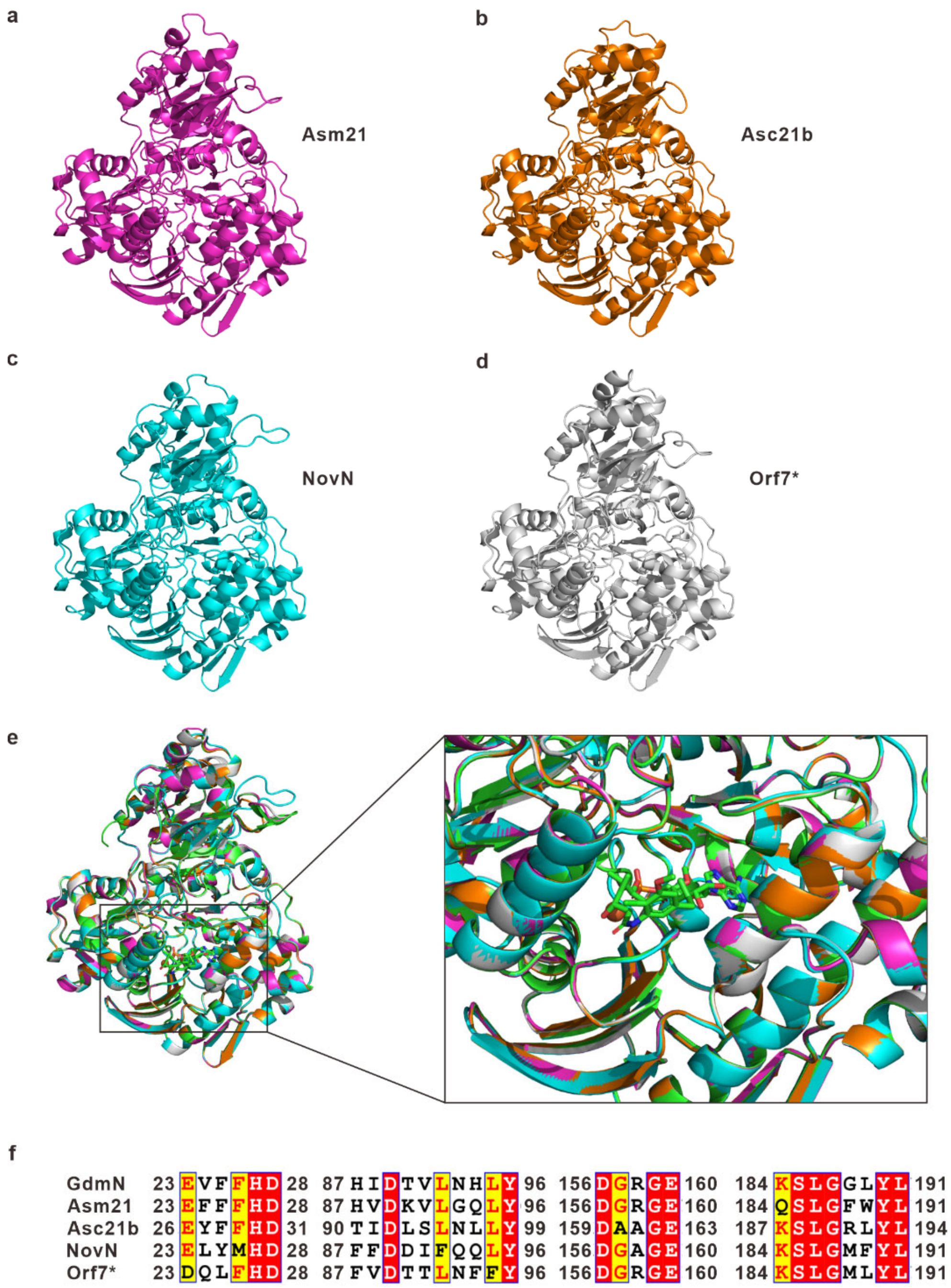


**Supplementary Figure 39 Monomer models, structural superposition, and sequence alignment for substrate binding of Asm21, Asc21b, NovN, and Orf7*.** **(a)**, **(b)**, **(c)** and **(d)** Monomer models of Asm21, Asc21b, NovN and Orf7* constructed by SWISS-MODEL server. **(e)** Superimposition of these models onto the GdmN/AMP/**1** complex. The dissection of the binding pocket indicated that these

CTases have evolved to similar broaden and relatively hydrophobic binding pocket. **(f)** Amino acid sequence alignment for substrate binding of GdmN-type CTases.

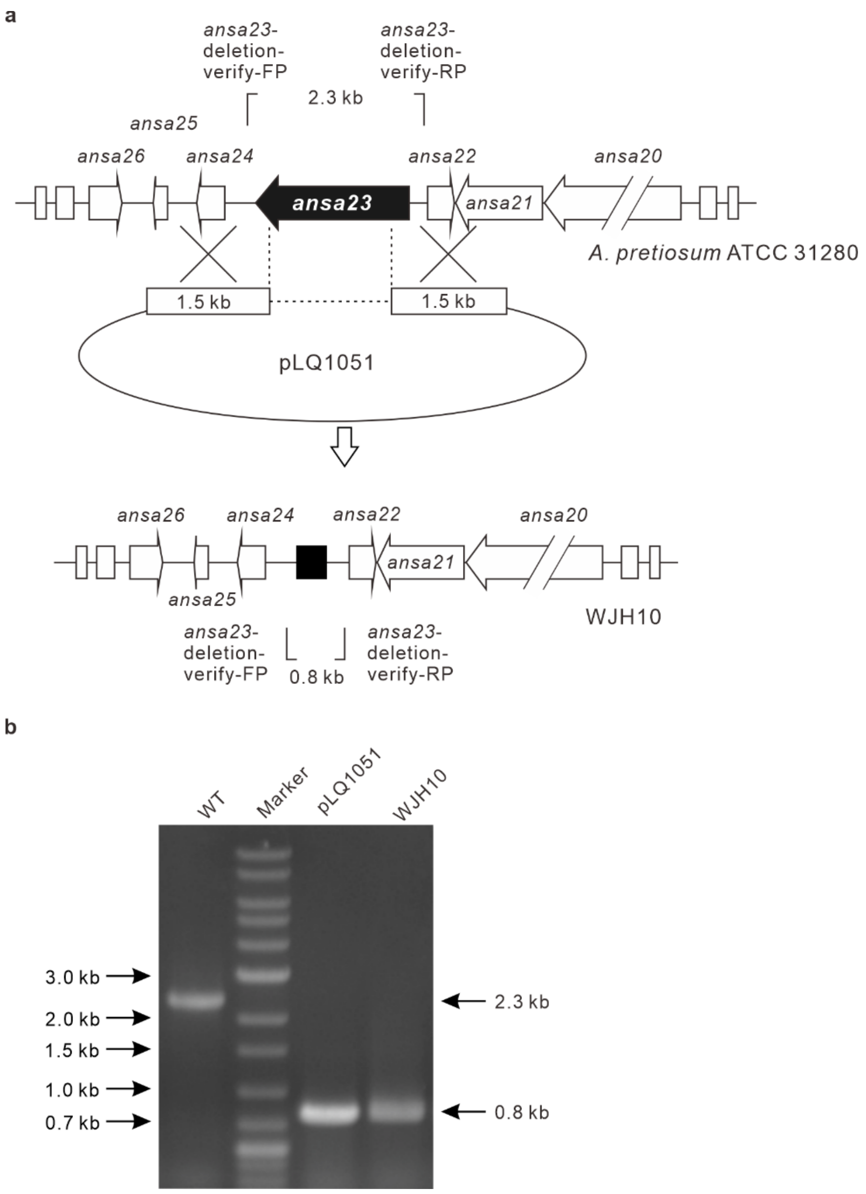


**Supplementary Figure 40 Schematic construction and PCR validation of WJH10.** **(a)** Schematic construction of *ansa23* mutant WJH10 by homologous recombination. **(b)** Gel electrophoresis of the PCR-amplified fragments using total genomic DNA of *A. pretiosum* ATCC 31280, pLQ1051, or the mutant strain WJH10 as templates. The PCR products of the mutant strain WJH10 and pLQ1051 were 0.8 kb, and the PCR product of *A. pretiosum* ATCC 31280 was 2.3 kb. The experiments were repeated for three times with similar results.

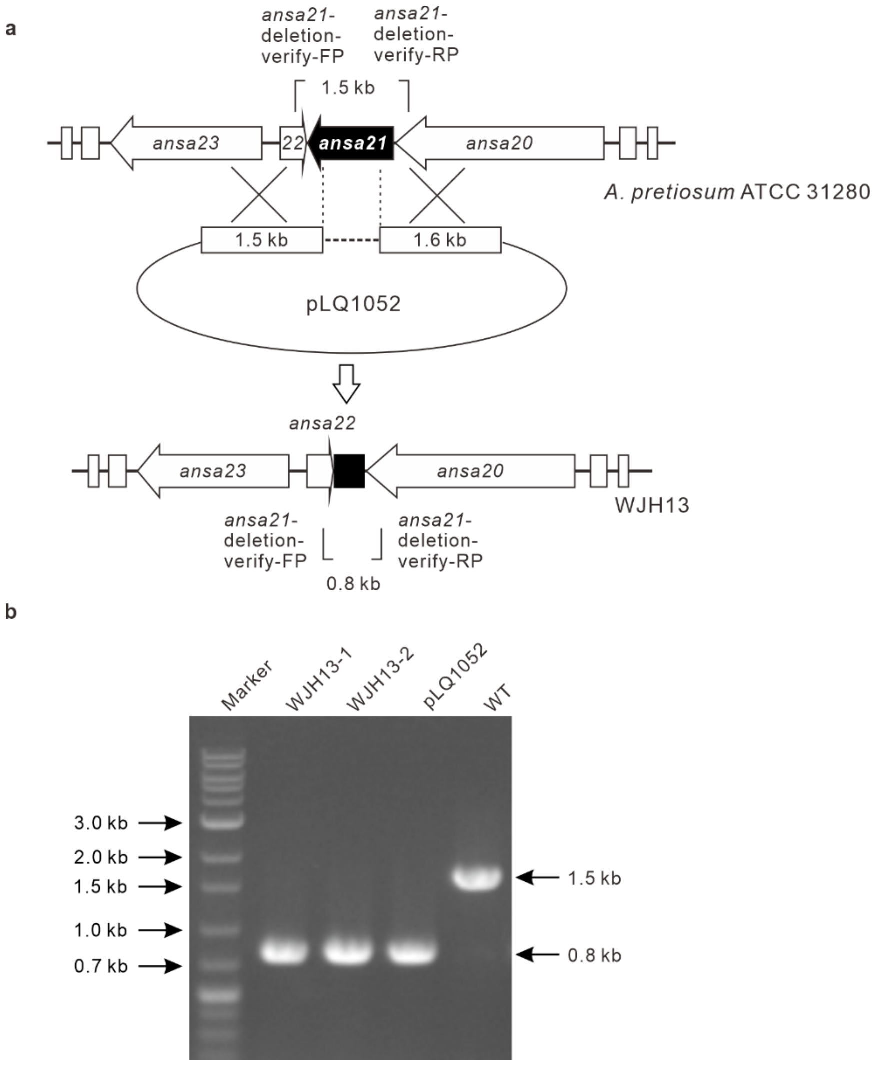


**Supplementary Figure 41 Schematic construction and PCR validation of WJH13.** **(a)** Schematic construction of *ansa21* mutant WJH13 by homologous recombination. (b) Gel electrophoresis of the PCR-amplified fragments using total genomic DNA of *A. pretiosum* ATCC 31280, pLQ1052, and the mutant strains WJH13 as templates. The PCR products of the mutant strains WJH13 and pLQ1052 were 0.8 kb, and the PCR product of *A. pretiosum* ATCC 31280 was 1.5 kb. The experiments were repeated for three times with similar results.

a

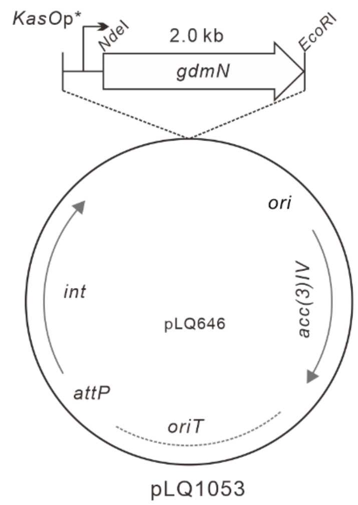


b

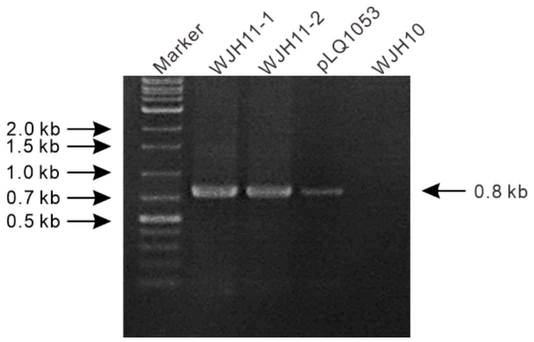


**Supplementary Figure 42 Schematic construction and PCR validation of WJH11.**

**(a)** Schematic construction of WJH11. **(b)** Gel electrophoresis of the PCR-amplified fragments using total genomic DNA of WJH10, pLQ1053, or the mutant strains WJH11 as templates. The PCR products of the mutant strains WJH11 and pLQ1053 were 0.8 kb, and there was no PCR product of WJH10. The experiments were repeated for three times with similar results.

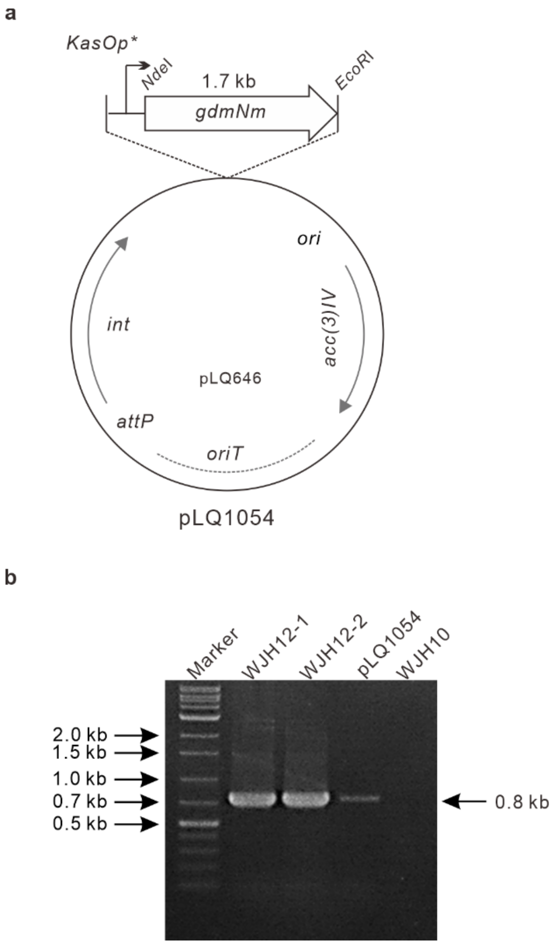


**Supplementary Figure 43 Schematic construction and PCR validation of WJH12.** **(a)** Schematic construction of WJH12. **(b)** Gel electrophoresis of the PCR-amplified fragments using total genomic DNA of WJH10, pLQ1054, or the mutant strains WJH12 as templates. The PCR products of the mutant strains WJH12 and pLQ1054 were 0.8 kb, and there was no PCR product of WJH10. The experiments were repeated for three times with similar results.

## Supplementary References


1. Wu, Z., Bai, L., Wang, M. & Shen, Y. Structure–antibacterial relationship of nigericin derivatives. *Chem. Nat. Compd.* **45**, 333-337 (2009).
2. Bierman, M. *et al.* Plasmid cloning vectors for the conjugal transfer of DNA from *Escherichia coli* to *Streptomyces* spp. *Gene* **116**, 43-49 (1992).
3. Kang, J.S., Son, B.W., Choi, H.D., Yoon, J.H. & Son, W.S. Dynamics of supercoiled and linear pBluescript II SK(+) Phagemids probed with a long-lifetime metal-ligand complex. *J. Biochem. Mol. Biol.* **38**, 104-110 (2005).
4. He, Y. *et al.* Two pHZ1358-derivative vectors for efficient gene knockout in *Streptomyces*. *J. Microbiol. Biotechnol.* **20**, 678-682 (2010).
5. Wang, X. *et al.* Efflux identification and engineering for ansamitocin P-3 production in *Actinosynnema pretiosum*. *Appl. Microbiol. Biotechnol.* **105**, 695-706 (2021).
6. Di Tommaso, P. *et al.* T-Coffee: a web server for the multiple sequence alignment of protein and RNA sequences using structural information and homology extension. *Nucleic Acids Res.* **39**, W13-17 (2011).
7. Robert, X. & Gouet, P. Deciphering key features in protein structures with the new ENDscript server. *Nucleic Acids Res.* **42**, W320-324 (2014).
