# Supplementary material for: Endowing homodimeric carbamoyltransferase GdmN with iterative functions through structural characterization and mechanistic studies
Source: Nat Commun. 2022 Nov 3;13:6617. doi: 10.1038/s41467-022-34387-2 (PMC9633730; doi:10.1038/s41467-022-34387-2)
Supplement: Supplementary file 3 — Reporting Summary [file 41467_2022_34387_MOESM3_ESM.pdf]

nature research

Corresponding author(s): Linquan Bai

Last updated by author(s): Oct 14, 2022

Reporting Summary

Nature Research wishes to improve the reproducibility of the work that we publish. This form provides structure for consistency and transparency in reporting. For further information on Nature Research policies, seeAuthors & Referees and theEditorial Policy Checklist .

Statistics

For all statistical analyses, confirm that the following items are present in the figure legend, table legend, main text, or Methods section.

n/a	Confirmed	
☐	☒	The exact sample size (n) for each experimental group/condition, given as a discrete number and unit of measurement
☐	☒	A statement on whether measurements were taken from distinct samples or whether the same sample was measured repeatedly
☐	☒	The statistical test(s) used AND whether they are one- or two-sided<br>*Only common tests should be described solely by name; describe more complex techniques in the Methods section.*
☒	☐	A description of all covariates tested
☒	☐	A description of any assumptions or corrections, such as tests of normality and adjustment for multiple comparisons
☐	☒	A full description of the statistical parameters including central tendency (e.g. means) or other basic estimates (e.g. regression coefficient) AND variation (e.g. standard deviation) or associated estimates of uncertainty (e.g. confidence intervals)
☐	☒	For null hypothesis testing, the test statistic (e.g. F, t, r) with confidence intervals, effect sizes, degrees of freedom and P value noted<br>*Give P values as exact values whenever suitable.*
☒	☐	For Bayesian analysis, information on the choice of priors and Markov chain Monte Carlo settings
☒	☐	For hierarchical and complex designs, identification of the appropriate level for tests and full reporting of outcomes
☒	☐	Estimates of effect sizes (e.g. Cohen's d, Pearson's r), indicating how they were calculated

Our web collection on statistics for biologists contains articles on many of the points above.

Software and code

Policy information about availability of computer code

Data collection	Crystal diffraction data sets of GdmN and GdmN complexes were collected at the BL18U1 and BL19U1 beamlines of the Shanghai Synchrotron Radiation Facility by using a DECTRIS PILATUS3 6M detector at a wavelength of 0.97853 Å at 100 K. The single-crystal X-ray diffraction data were collected on a Bruker D8 VENTURE CMOS Photon II diffractometer with helios mx multilayer monochromator Cu Kα radiation (λ = 1.54178 Å) at 173 K.
Data analysis	Diffraction data were processed and scaled using the HKL3000 or HKL2000 program. Structures of GdmN and GdmN complexes were determined by molecular replacement using the program Phaser (version 2.7.17) from PHENIX(version 1.19.2-4158). Iterative cycles of model rebuilding and refinement were performed using COOT (version 0.9.4) and PHENIX, to generate the final model of GdmN. PyMOL (version 2.3.1), Gaussian 16, GraphPad Prism version 9.0.2, AMBER 2018, ChemShell 3.6.0, Turbomole 7.2, VMD 1.9.1, MestReNova 9.0.1, APEX3 v2019.11-0, SHELXL-2018/3 program package, MEGA 11

For manuscripts utilizing custom algorithms or software that are central to the research but not yet described in published literature, software must be made available to editors/reviewers. We strongly encourage code deposition in a community repository (e.g. GitHub). See the Nature Research guidelines for submitting code & software for further information.

Data

Policy information about availability of data

All manuscripts must include a data availability statement. This statement should provide the following information, where applicable:

- Accession codes, unique identifiers, or web links for publicly available datasets
- A list of figures that have associated raw data
- A description of any restrictions on data availability

Data Availability
Data supporting the findings of this work are available within the paper and its Supplementary Information files. A reporting summary for this Article is available as

a Supplementary Information file. X-ray crystallographic coordinates have been deposited in the Protein Data Bank (PDB) with the accession codes 7VYO [http://10.2210/pdb7vyo/pdb], 7VX0 [http://10.2210/pdb7vx0/pdb], 7VYJ [http://10.2210/pdb7vyj/pdb], 7VZY [http://10.2210/pdb7vzy/pdb], 7VZN [http://10.2210/pdb7vzn/pdb], 7VZZ [http://10.2210/pdb7vzz/pdb], 7VYP [http://10.2210/pdb7vyp/pdb], 7VZU [http://10.2210/pdb7vzu/pdb], and 7VZQ [http://10.2210/pdb7vzq/pdb]. The X-ray crystallographic data for 4 have been deposited at the Cambridge Crystallographic Data Centre with the accession number CCDC 2122658 [https://doi.org/10.5517/ccdc.csd.cc297sv6]. Copies of the data can be obtained free of charge via https://www.ccdc.cam.ac.uk/structures/. Source data are provided with this paper.

Field-specific reporting

Please select the one below that is the best fit for your research. If you are not sure, read the appropriate sections before making your selection.

☒ Life sciences ☐ Behavioural & social sciences ☐ Ecological, evolutionary & environmental sciences

For a reference copy of the document with all sections, see nature.com/documents/nr-reporting-summary-flat.pdf

Life sciences study design

All studies must disclose on these points even when the disclosure is negative.

Sample size	No sample size calculations were performed for this study. Sample sizes are indicated for each experiment and were chosen based on similar studies. Experiments were performed in triplicates (n = 3).
Data exclusions	No data were excluded from the analyses.
Replication	All experiments were performed in triplicates and all attempts at replication were successful.
Randomization	Because there were no need to allocate samples into experimental groups in essence, this is not relevant to our study.
Blinding	Because there were no need to allocate samples into experimental groups in essence, this is not relevant to our study.

Reporting for specific materials, systems and methods

We require information from authors about some types of materials, experimental systems and methods used in many studies. Here, indicate whether each material, system or method listed is relevant to your study. If you are not sure if a list item applies to your research, read the appropriate section before selecting a response.

Materials & experimental systems

n/a	Involved in the study	
☒	☐	Antibodies
☒	☐	Eukaryotic cell lines
☒	☐	Palaeontology
☒	☐	Animals and other organisms
☒	☐	Human research participants
☒	☐	Clinical data

Methods

n/a	Involved in the study	
☒	☐	ChIP-seq
☒	☐	Flow cytometry
☒	☐	MRI-based neuroimaging
